# Genome-Wide Identification of *Chalcone Reductase* Gene Family in Soybean: Insight into Root-Specific *GmCHR*s and *Phytophthora sojae* Resistance

**DOI:** 10.3389/fpls.2017.02073

**Published:** 2017-12-07

**Authors:** Caroline J. Sepiol, Jaeju Yu, Sangeeta Dhaubhadel

**Affiliations:** ^1^London Research and Development Centre, Agriculture and Agri-Food Canada, London, ON, Canada; ^2^Department of Biology, University of Western Ontario, London, ON, Canada

**Keywords:** soybean, chalcone reductase, gene family, *Phytophthora sojae*, root and stem rot disease, resistance, quantitative trait loci, plant stress

## Abstract

Soybean (*Glycine max* [L.] Merr) is one of the main grain legumes worldwide. Soybean farmers lose billions of dollars’ worth of yield annually due to root and stem rot disease caused by the oomycete *Phytophthora sojae*. Many strategies have been developed to combat the disease, however, these methods have proven ineffective in the long term. A more cost effective and durable approach is to select a trait naturally found in soybean that can increase resistance. One such trait is the increased production of phytoalexin glyceollins in soybean. Glyceollins are isoflavonoids, synthesized via the legume-specific branch of general phenylpropanoid pathway. The first key enzyme exclusively involved in glyceollin synthesis is chalcone reductase (CHR) which coacts with chalcone synthase for the production of isoliquiritigenin, the precursor for glyceollin biosynthesis. Here we report the identification of 14 putative *CHR* genes in soybean where 11 of them are predicted to be functional. Our results show that *GmCHR*s display tissue-specific gene expression, and that only root-specific *GmCHR*s are induced upon *P. sojae* infection. Among 4 root-specific *GmCHR*s, *GmCHR2A* is located near a QTL that is linked to *P. sojae* resistance suggesting *GmCHR2A* as a novel locus for partial resistance that can be utilized for resistance breeding.

## Introduction

Soybean (*Glycine max* [L.] Merr) is one of the most important grain legumes in the world. The seeds are an excellent source of protein, oil, micronutrients and specialized metabolites such as isoflavonoids and saponins, making it a profitable crop for human or livestock consumption and many industrial products. From this versatility, the United States Department of Agriculture has estimated that the Global Soybean Production in 2017/2018 is 348.04 million metric tons, which is an increase of 11.18% in soybean production worldwide^1^. In Canada, soybean was the 4th largest crop grown in 2016, producing 6.46 million metric tons and generating $2.4 billion in profits^2^. Though these numbers appear promising, soybean farmers encounter about $50 million of soybean yield loss annually in Canada, and $1–2 billion worldwide due to stem and root rot disease caused by *Phytophthora sojae*.

The traditional strategies to reduce the incidence of *P. sojae* infection such as soil treatment with calcium application (Sugimoto et al., 2010), fungicide application (Anderson and Buzzell, 1982) and improved soil drainage and tillage (Workneh et al., 1998) have not only proven to be ineffective, but also found to place selective pressures on *P. sojae* leading to resistance (Li et al., 2010). An alternative approach to this problem is selecting a cultivar of soybean with optimal resistance to *P. sojae* infection. Resistance to pathogen infection in soybeans can either be complete or partial. Complete resistance or race-specific resistance is conferred by single dominant resistance to *P. sojae* (*Rps*) genes, which counteract the virulence genes within *P. sojae*. This relationship is similar to effector-triggered immune response in other pathosystems (Sugimoto et al., 2012). To date, there are 27 *Rps* genes and more than 200 identified races of *P. sojae* (Sahoo et al., 2017). However, the rapid evolution of *P. sojae* races continues to diversify and as a result the new races become ineffective to the *Rps* genes leading to disease susceptibility. Partial resistance, referred to as field resistance, is more durable and a broad-spectrum non-race-specific trait conferred by several minor genes which involves various defense components. Cultivars with this type of resistance contain fewer damaged roots than completely susceptible cultivars, show delayed disease progression, and are effective against all races of *P. sojae* (Schmitthenner, 1985). Some key traits such as increased levels of soybean root suberin and increased production of phytoalexin glyceollins have been correlated with strong partial resistance in soybean (Thomas et al., 2007; Lygin et al., 2013). Isoflavonoids act as phytoalexins in soybean that play a role as plant’s basal and innate response to biotic and abiotic stress. When isoflavonoid production was compromised by down regulating the biosynthetic genes, *chalcone reductase* (*CHR*) and *isoflavone synthase* (*IFS*), it reduced the plants’ ability to fight off the pathogen attack (Subramanian et al., 2005; Graham et al., 2007; Lozovaya et al., 2007).

**FIGURE 1:**
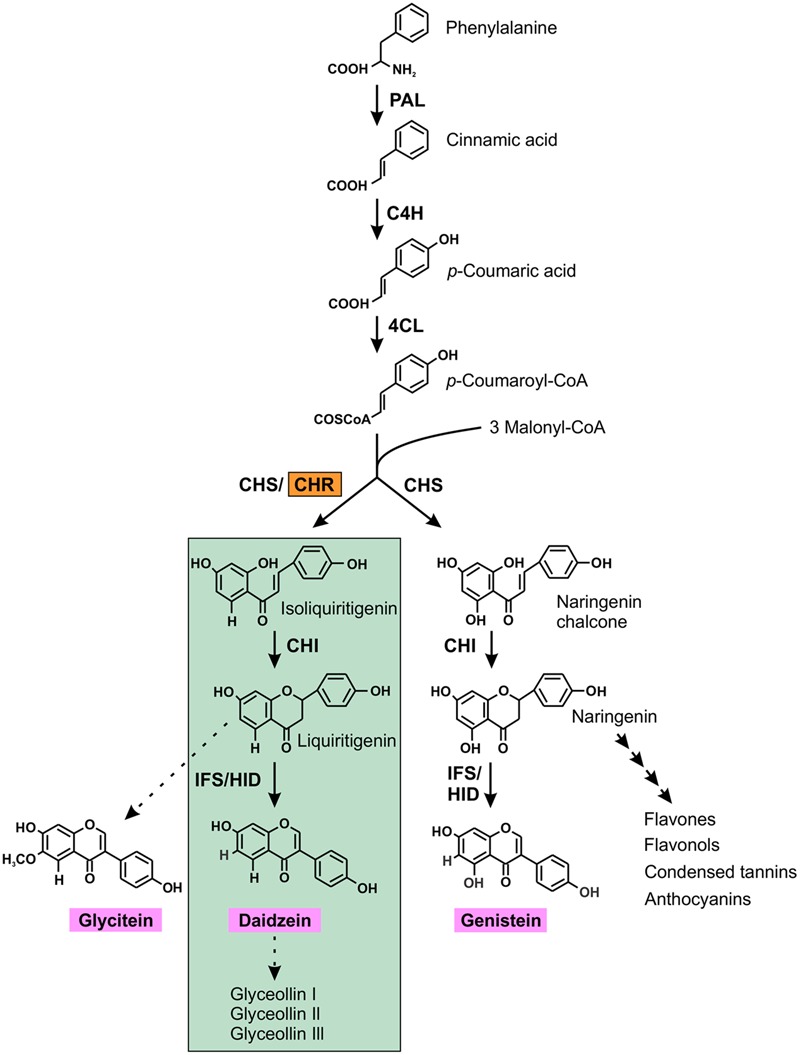
(Iso) flavonoid biosynthetic pathway in soybean. The first key legume-specific enzyme chalcone reductase (CHR) is highlighted in orange that leads into the synthesis of isoflavonoid phytoalexins (green highlights) in soybean. The three isoflavone aglycones glycitein, daidzein and genistein are shown in pink. The dotted arrow indicates speculative steps while multiple arrows indicate two or more steps in the pathway. PAL, phenylalanine ammonia lyase; C4H, cinnamate 4-hydroxylase; 4CL, 4-coumarate:CoA ligase; CHR, chalcone reductase; CHS, chalcone synthase; CHI, chalcone isomerase; IFS, isoflavone synthase; 2HID, 2-hydroxyisoflavanone dehydratase.

CHR (formally known as polyketide reductase) is the key enzyme for isoflavone aglycone daidzein biosynthesis which ultimately leads to the production of the phytoalexin glyceollins in soybean (Dakora and Phillips, 1996). The isoflavonoid pathway begins with the general phenylproponoid pathway that branches off into two sub-branches: (i) chalcone synthase (CHS) producing naringenin chalcone, a compound that subsequently synthesizes genistein, a core isoflavone aglycone and/or many other flavonoids; (ii) CHS coacting with CHR producing isoliquiritigenin, the building block of the other two core isoflavone aglycones, glycitein and daidzein (**Figure 1**).

Chalcone reductase belongs to the aldo-keto reductase (AKR) sub-family 4 in the large AKR superfamily (Jez et al., 1997). All members of this superfamily fold into a monomeric, (α/β)_8_ barrel structure and contain a catalytic tetrad of Asp-53, Tyr-58, Lys-87, and His-120 and a common NAD(P)(H) binding site that is located in a deep, large and hydrophobic pocket at the C-terminus end (Bomati et al., 2005). All CHRs predominantly contain hydrophobic and aromatic residues that line the unoccupied entrance to the active site cavity molded by Pro-29, Ala-57, Trp-89, Phe-130, and Phe-132. Largely polar residues define the base of this catalytic surface and include the catalytic tetrad, Trp-121 and Asn-167 (Bomati et al., 2005). Beside these facts, very little is known about CHR enzyme since it acts on intermediates for CHS, and identity of its substrate still remains unknown.

The first CHR activity was discovered in the crude extracts of *Glycyrrhiza echinata* (Ayabe et al., 1988). Up to now, CHR-like enzymes have been identified in a variety of leguminous plant species, including *Medicago sativa* (Ballance and Dixon, 1995), *Sesbania rostrata* (Goormachtig et al., 1999), *Pueraria montana* var. lobata (He et al., 2011), *Glycyrrhiza glabra* (Hayashi et al., 2013), and *Lotus japonicus* (Shimada et al., 2006). Graham et al. (2007) identified 4 soybean *CHR*s through the Expressed Sequence Tag (EST) database search^3^. RNAi silencing of these *CHRs* in soybean hairy roots resulted into reduced levels of isoflavonoids and increased susceptibility to *P. sojae* infection compared to the control (Graham et al., 2007).

Here, we performed a genome-wide search of *AKR* gene family members and identified 14 *CHR* genes in soybean. The phylogenetic history of the GmCHRs, and their sequence analysis suggested 11 GmCHRs as catalytically active for its role. Transcript analysis revealed that 4 root-specific *GmCHR*s are induced upon stress. Some of the stress induced *GmCHR*s locate in the QTL regions that are linked to *P. sojae* resistance. These *GmCHR* genes together with other minor genes and *Rps* gene can be stacked in the same cultivar for future resistant breeding.

## Materials and Methods

### Plant Materials and Growth Conditions

Seeds of soybean (*Glycine max* L. Merr.) cv Harosoy63, OX760-6 and Conrad, and *Nicotiana benthamiana* were grown in a growth room with 16 h light at 23°C and 8 h dark cycle at 18°C, with 60–70% relative humidity and a light intensity of 100–150 μmol m^-2^ s^-1^.

### *In Silico* Analysis

To identify all *CHR* gene family members in the soybean genome, a search was conducted in the annotated *G. max* Wm82.a2.v1 genome of Phytozome^4^. The keywords “aldo-keto” and “aldo/keto” were used to find all the soybean aldo-keto reductases (GmAKRs). To ensure no *GmAKRs* were missed in the keyword search, each *GmAKR* was used as a query for a BLAST search again. For generating a phylogenetic tree, protein sequences were aligned using CLUSTALO^5^ and a Neighbor-joining tree based with 1000 bootstrap replications was created using MEGA7 (Kumar et al., 2016). The Poisson method was selected to calculate the evolutionary distance of the phylogenetic tree and pairwise deletion was selected for gaps/missing data treatment. To determine whether all candidate GmCHRs contain residues deemed important for catalytic activity, the protein sequences of the candidate *GmCHR* were aligned using CLUSTALO followed by BOXSHADE 3.21^6^. Critical residues were manually spotted based on Bomati et al. (2005).

The QTLs and QTL markers from the year 2003 to 2016 corresponding to *P. sojae* resistance were extracted from the SoyBase and Soybean Breeder’s Toolbox^7^. To ensure no QTLs or QTL marker were missed in the search; a literature search was also conducted. Relative positions of *GmCHRs*, and QTL markers were mapped onto the chromosomes. QTLs regions in base pairs were noted from the *G. max* genome assembly on Soybase.org.

### RNA Extraction, cDNA Synthesis, RT/qRT-PCR and Gene Expression Analysis

Total RNA was extracted from 100 mg of tissue using the RNeasy Plant Mini Kit (Qiagen) following manufacturer’s instruction with some modification. An on-column DNaseI (Promega) treatment was used to digest DNA. Thermoscript RT-PCR System (Life Technologies) was used to synthesize cDNA from total RNA. PCR amplification was performed using gene-specific primers (Supplementary Table S1). SsoFast EvaGreen Supermix Kit (BioRad) was used for qPCR reaction with gene-specific primers (Supplementary Table S1). *CONS4* was used as a reference gene to normalize the expression (Libault et al., 2008).

A publicly available RNA-seq database containing transcriptome sequencing of soybean^8^ was mined for the tissue-specific expression profiles of *GmCHR* gene family members. The relative expression was normalized across the libraries corresponding to each tissue. A heatmap for *GmCHR* transcripts was generated in R.

### Western Blot Analysis

Samples (0.5 g) were ground in liquid nitrogen and re-suspended in protein extraction buffer (25 mM Tris-HCL pH 8.0, 1 mM EDTA pH 8.0, 20 mM NaCl) supplemented with protease inhibitor cocktail (Sigma–Aldrich) for protein extraction. Total soluble proteins (30 μg) were separated on a SDS-PAGE, transferred onto PVDF membrane (Bio-Rad) using a Trans-Blot Semi-Dry Electrophoretic Transfer Cell (Bio-Rad). The fusion proteins with YFP were detected using an anti-GFP (1:7000 dilution) mouse primary antibody and conjugated horseradish peroxidase goat anti-mouse (1:5000 dilution) secondary antibody, followed by treatment with ECL Prime Western Blot detection reagents (GE Health Care Life Sciences).

### Stress Treatment

Seven-day old seedlings of soybean cv. L76-1988 were inoculated with *P. sojae* race 7. The stems of the infected plants were collected at 24, 48, and 72 h post-inoculation. For the AgNO_3_ treatment, soybean cv. Harosoy63 was grown in water-soaked vermiculite in the dark at 25°C for 6 days. Prior to the treatment, 10 etiolated seedlings per treatment were transferred into glass trays, after which, 5–10 drops of 10 μL of either water (control) or 1 mM of AgNO_3_ were placed onto the hypocotyl of each seedling (**Supplementary Figure S1**). The trays were transferred back in the dark to 25°C. Samples were collected at 6, 12, 24, 48, or 72 h after the treatment.

### Plasmid Construction and Subcellular Localization

For subcellular localization study, *GmCHR*s were amplified by PCR using gene-specific primers (Supplementary Table S1) and cloned into the gateway entry vector pDONR-Zeo (Invitrogen) using the BP clonase reaction mix (Invitrogen), transformed into *Escherichia coli* DH5α. The recombinant plasmid pDONR-zeo-GmCHR was recombined with the destination vector pEarlyGate101 using the LR clonase reaction mix (Invitrogen). The sequence confirmed pEarlyGate101 was transformed into *Agrobacterium tumefaciens* GV3101 via electroporation.

The constructs in *A. tumefaciens* GV3101 were transformed into *N. benthamiana* leaves by infiltration (Sparkes et al., 2006), and transient expression was visualized through a Leica TCS SP2 inverted confocal microscope. To confirm the nuclear localization of the proteins, *A. tumefaciens* GV3101 with the construct containing nuclear localization signal fused to CFP (pEarleyGate100-NLS-CFP) and pEarleyGate101-GmCHR were mixed 1:1 and infiltrated into the leaves followed by confocal microscopy. For YFP visualization, an excitation wavelength of 514 nm was used and emissions were collected between 525 and 545 nm. For visualization of CFP, an excitation wavelength of 434 nm was used and emissions were collected between 460 and 490 nm.

## Results

### The Soybean Genome Contains 14 Putative *GmCHR*s

Since CHRs belong to the AKR superfamily, we performed a keyword search in the annotated *G. max* Wm82.a2.v1 genome on Phytozome to identify all the GmAKRs. Using the keywords “aldo-keto” and “aldo/keto”, protein databases KOG, Pfam and PANTHER identified 34 and 67 *GmAKRs*, respectively. Results from both keyword searches were compared and compiled to ensure no duplicates. Each *GmAKR* sequence was then used as a query for a BLAST search in *G. max* Wm82.a2.v1 genome, until no new *GmAKRs* were identified. This process identified a total of 67 unique *AKR* genes in soybean.

**FIGURE 2:**
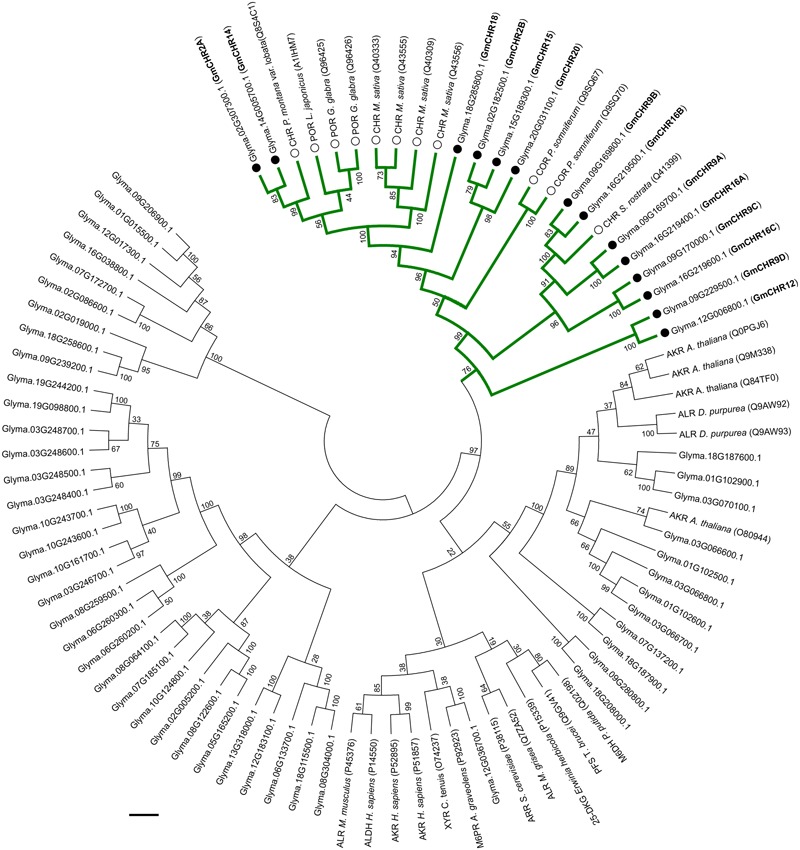
Phylogenetic analysis of GmAKRs and GmCHRs. The deduced amino acid sequences of putative GmAKRs and known AKRs from other plant species were aligned using CLUSTALO and the Neighbor-joining tree was constructed using MEGA7 software. The green branch indicates CHR-specific AKRs, black circles (●) and white circles (○) indicate putative GmCHRs and CHRs from other plants species, respectively. POR, polyketide reductase; COR, codeinone reductase; AKR, aldo-keto reductase; XYR, xylose reductase; M6PR, mannose-6-phosphate reductase; M6DH, morphine 6-dehydrogenase; ALHD, alcohol dehydrogenase; ALR, aldose reductase; ARR, arabinose reductase; 2,5 DKG, 2,5-diketo-D-gluconic acid reductase B; and PFS, prostaglandin F synthase. Common nomenclature for GmCHRs is also shown in parenthesis (bold).

The AKR superfamily consists of 16 families; where CHRs from different plant species group into the AKR4 subfamily (Jez et al., 1997; Penning, 2015). To identify GmCHRs in soybean, a phylogenetic analysis was performed using the 67 candidate GmAKRs and previously characterized AKRs from Bomati et al. (2005) with the assumption that the GmCHRs would cluster together with the known CHRs from other plant species. As shown in **Figure 2**, 14 putative soybean CHRs clustered together with known CHRs from other plant species. GmCHR2A, GmCHR14, GmCHR18, GmCHR2B, GmCHR15 and GmCHR20 clustered closely with CHRs from *M. sativa* and *P. montana* var. lobata, POR from *L. japonicus* and *G. glaba*. Other inter-species clustering consisted of GmCHR9B, GmCHR16B, GmCHR9A, GmCHR16A, GmCHR9C, GmCHR16C, GmCHR9D, and GmCHR12 with CHR from *S. rostrata*. Codeinone reductase, a non-CHR, is also found in the same branch as CHR, however, none of the GmCHRs cluster together with this group.

**FIGURE 3:**
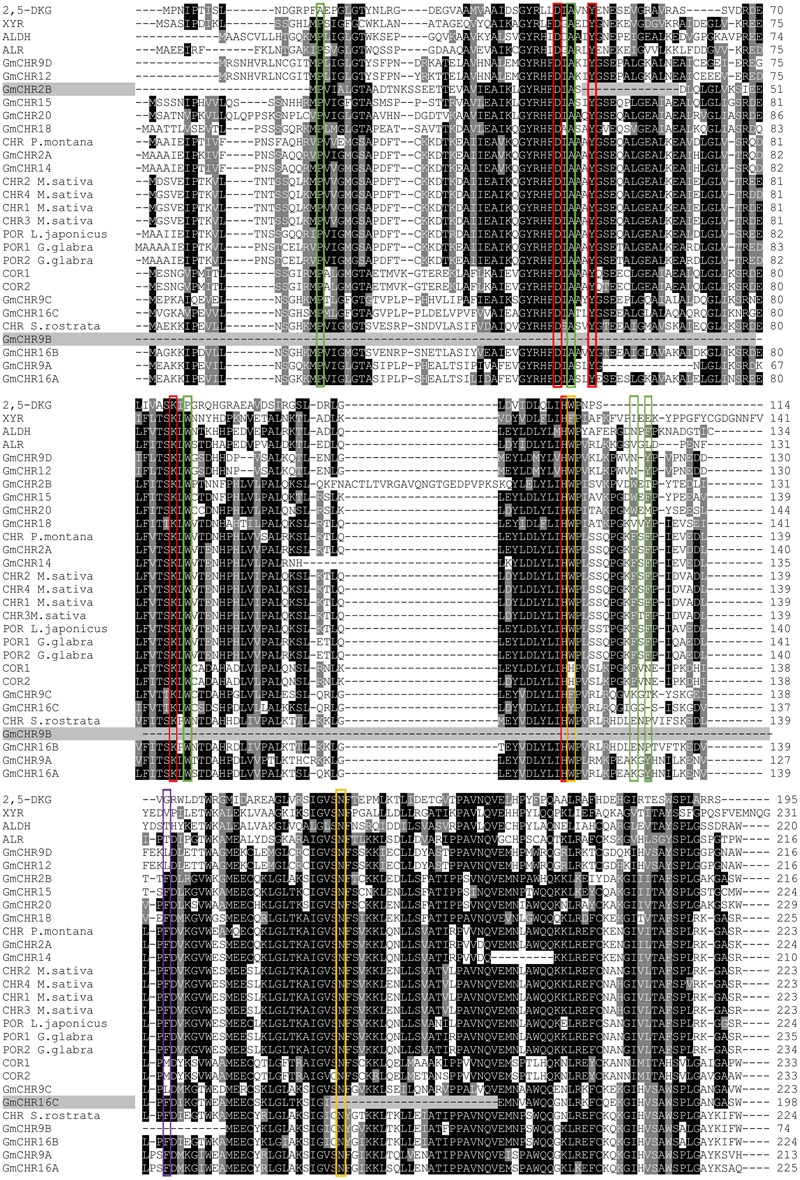
Identification of important amino acid residues in putative GmCHRs. Candidate GmCHRs obtained from the phylogenetic analysis were aligned using CLUSTALO with other known AKRs and CHRs from other plant species. Critical and other residues were noted: entrance of the catalytic site (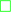), AKR catalytic tetrad (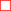), unique amino acids from COR (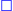), CHR active site with AKR catalytic tetrad (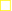). GmCHRs which are missing critical residue are indicated through gray shading. Only an abridged version of the alignment is shown.

The active site of CHR is primarily defined by the AKR family “catalytic tetrad” (Asp-53, Tyr-58, Lys-87, and His-120) with the additional polar residues Trp-121, and Asn-167 (Bomati et al., 2005). To identify whether all the putative GmCHRs contain critical amino acid residues that are required for CHR function, the amino acid sequences of the 14 putative GmCHRs were aligned with the known CHRs from other plant species, and a selection of non-CHR AKRs and CHR-specific active site residues were searched manually. This process identified the following alterations in 3 candidate GmCHRs: (1) in GmCHR2B, Tyr-58 was missing; (2) in GmCHR9B, Asp-53, Tyr-58, Lys-87, His-120, and Trp-121 were missing; and (3) Asn-167 was not present in GmCHR16C (**Figure 3**). Based on these results, GmCHR2B, GmCHR9B, and GmCHR16C were eliminated for further study as they lack one or more critical residues for their function.

Detailed information on the remaining 11 putative *GmCHRs*, including gene location, coding sequence length, protein molecular mass and predicted subcellular localization is shown in **Table 1**. Except for GmCHR12 and GmCHR14, all other GmCHRs were predicted to localize in the cytoplasm. Pairwise amino acid sequence identity comparison of the 11 *GmCHR* gene family members ranged from 50 to 97%. However, pairwise nucleotide sequence identity comparison ranged from 37 to 96% (**Table 2**).

**Table 1:** Characteristics of soybean *chalcone reductase* gene family.

Name gene	Locus name	Locus range	Splice variants	Coding sequence (nt)	Protein molecular weight (kDa)	Predicted sub-localization
*GmCHR2A*	Glyma.02G307300	Chr02: 48163443..48164865	1	948	35.59	Cytoplasm
*GmCHR9A*	Glyma.09G169700	Chr09: 39437065..39439064	1	846	31.86	Cytoplasm
*GmCHR9C*	Glyma.09G170000	Chr09: 39453516..39456085	1	957	35.48	Cytoplasm
*GmCHR9D*	Glyma.09G229500	Chr09: 45341437..45343797	1	969	36.49	Cytoplasm
*GmCHR12*	Glyma.12G006800	Chr12: 508878..511003	1	948	35.85	Chloroplast
*GmCHR14*	Glyma.14G005700	Chr14: 463009..64543	1	981	36.77	Nucleus
*GmCHR15*	Glyma.15G189300	Chr15: 20075936..20082802	1	948	35.43	Cytoplasm
*GmCHR16A*	Glyma.16G219400	Chr16: 37672779..37675776	3	963	36.15	Cytoplasm
*GmCHR16B*	Glyma.16G219500	Chr16: 37677371..37670638	1	969	36.04	Cytoplasm
*GmCHR18*	Glyma.18G285800	Chr18: 56611460..56612978	2	948	35.14	Cytoplasm
*GmCHR20*	Glyma.20G031100	Chr20: 3790362..3793599	1	966	35.91	Cytoplasm

*CHR, chalcone reductase; nt, nucleotide; kDa, kilo-Dalton.*

**Table 2:** Pairwise coding DNA and amino acid sequences in *chalcone reductase* gene family in soybean.

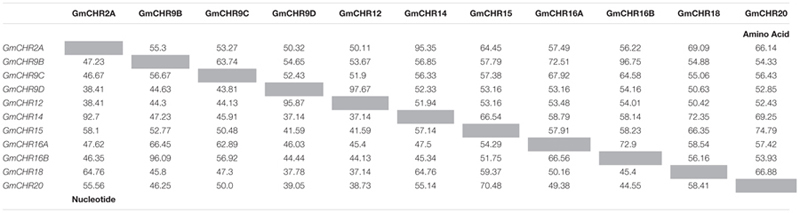

### *GmCHRs* Family Members Display Tissue-Specific Gene Expression

**FIGURE 4:**
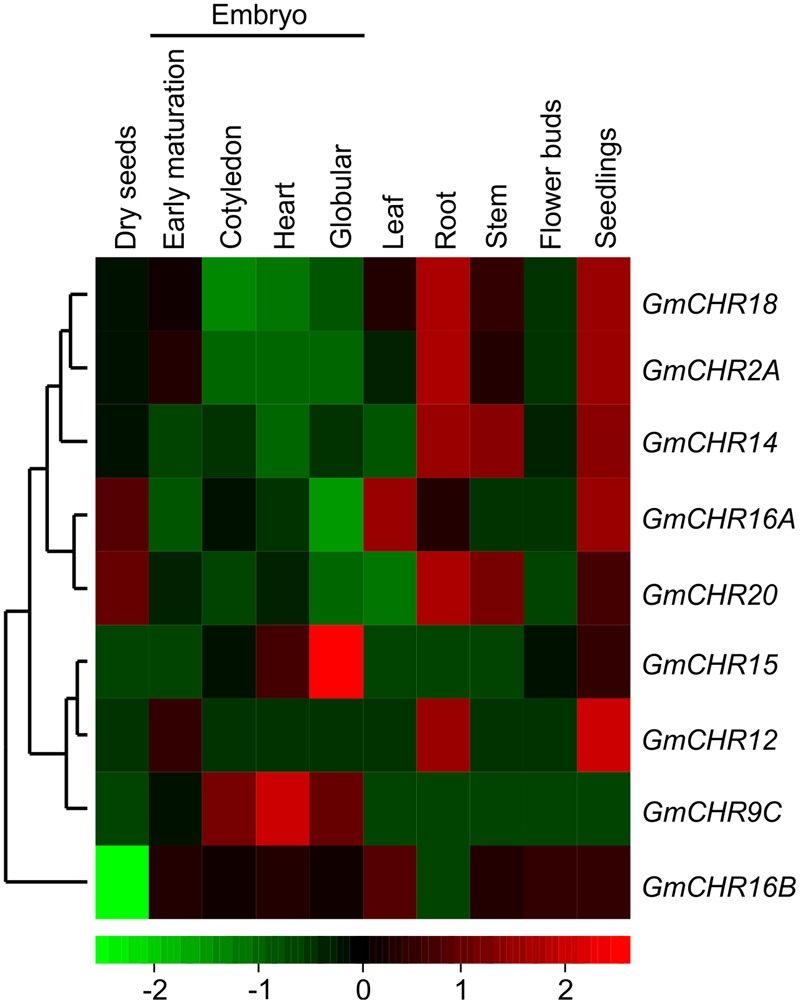
Tissue-specific expression profile of *CHR* genes in soybean. Soybean RNA-seq data across different tissues were retrieved from the National Center for Biotechnology Information (http://www.ncbi.nlm.nih.gov/geo/query/acc.cgi?acc=GSE29163) for heat map construction. Transcript abundance is indicated by the color scale below the heat map with a gradient from red (high) to green (low).

To determine tissue-specific expression pattern of *GmCHRs*, publicly accessible RNA-seq data derived from high throughput sequencing using total RNA isolated from various soybean tissues at different developmental stages such as developing embryos, mature seed, and a number of vegetative tissues^9^ was retrieved. The relative expression was normalized across the libraries corresponding to each tissue and a heatmap was produced based on the expression level of each *GmCHR* for each tissue. Out of 11 putative *GmCHRs, GmCHR2A, GmCHR14, GmCHR18, GmCHR12* and *GmCHR20* were expressed at higher level in roots as compared to other tissues (**Figure 4**). Majority of the *GmCHRs* were expressed in the seedlings except for *GmCHR9C*. Among the 5 root-specific *GmCHR*s, *GmCHR14* and *GmCHR20* transcript also accumulated in stem and seedling tissues. Transcript levels of *GmCHR15* and *GmCHR9C* were abundant in early embryo development indicating their role in embryo development. Tissue-specific transcript accumulation data for *GmCHR9A* and *GmCHR9D* could not be obtained from the RNAseq database^8^, therefore, they are not included in **Figure 4**. It is possible that these genes may not be expressed in whole seeds at various stages of development (globular, heart, cotyledon, early-, mid- and late-maturation, dry), leaf, root, stem, floral bud and seedling included in the study (Danzer et al., 2015).

### Root-Specific *GmCHRs* Are Induced upon Stress

**FIGURE 5:**
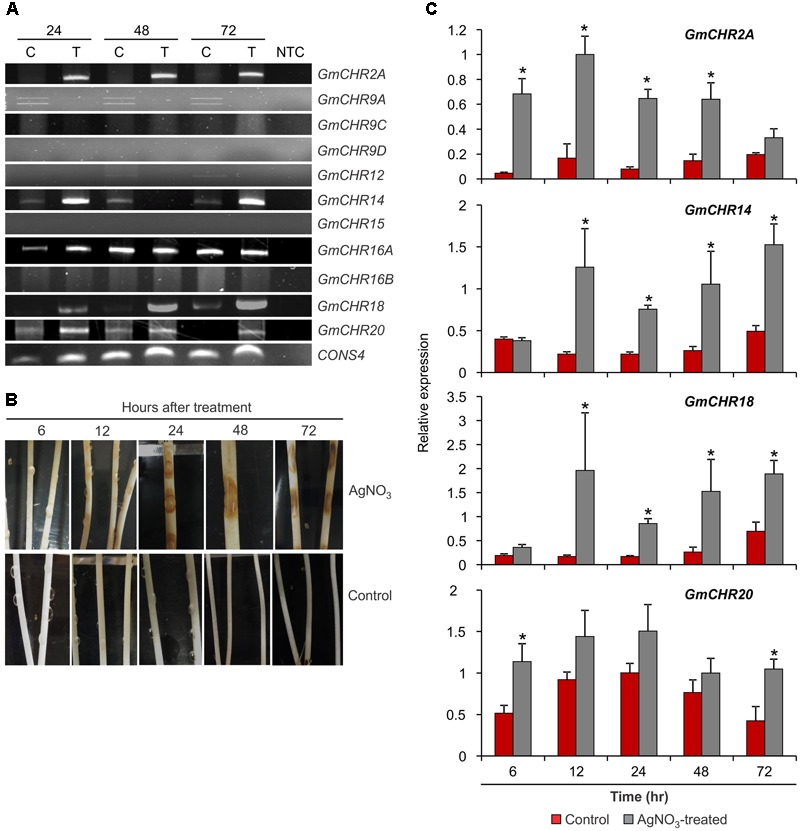
Expression of root-specific *GmCHR*s in response to stress. **(A)** Expression of *GmCHRs* in response to *P. sojae* infection. Total RNA (1 μg) from *P. sojae*-infected stems of soybean cv. L76-1988 (T) and control (C) for 24, 48 or 72 hr was used to synthesize cDNA. Expression analysis was conducted by RT-PCR with *GmCHR* gene-specific primers. NTC indicates no template control. *CONS4* was used as a loading control. **(B)** Effects of AgNO_3_ on etiolated soybean cv. Harosoy63 hypocotyls. Soybean seeds were grown in dark at 24°C for 6 days in water-soaked vermiculite. The seedlings were then placed onto a tray and inoculated with either water (control) or 1 mM AgNO_3_ (treated). Photographs were taken at the time points as indicated. **(C)** Detail expression analysis of 4 root-specific *GmCHRs*. Total RNA (1 μg) of soybean cv. Harosoy63 was used to synthesized cDNA from untreated and AgNO_3_-treated hypocotyls. Expression analysis was conducted by qPCR with *GmCHR* gene-specific primers. Error bars indicates standard error of the mean (SEM) of two biological and three technical replicates per biological replicates. *CONS4* was used as a reference gene. The asterisks (^∗^) denotes significant expression as determined by Student’s *t*-test (*p* < 0.05).

To identify *GmCHR* genes that are induced upon pathogen attack, stems of soybean cv. L76-1988 were infected with *P. sojae* agar culture. Stem samples were collected at 24, 48, and 72 h post-infection, and expression analysis of 11 putative *GmCHRs* was performed using RT-PCR with gene-specific primers. As shown in **Figure 5A**, expression of 4 root-specific *GmCHR* genes, *GmCHR2A, GmCHR14, GmCHR18* and *GmCHR20*, were induced after 24 h and remained induced until 72 h post-infection except for *GmCHR14* where no transcript accumulation was observed at 48 hr in treated tissue. Even though *GmCHR12* was expressed in root (**Figure 4**), it did not show induced expression upon *P. sojae* infection. Expressions of *GmCHR9C, GmCHR12* and *GmCHR16B* were also undetectable in both control and infected samples. However, accumulation of *GmCHR16A* remained unchanged in both infected and control samples.

Since AgNO_3_ treatment induces defense responses and phytoalexin production in soybean, it has been used in the past to mimic the effect of pathogen attack (Ward et al., 1979; Bhattacharyya and Ward, 1986; Moy et al., 2004; Kubeš et al., 2014). For quantitative analysis of stress-induced *GmCHR* expression in soybean, we treated etiolated soybean (cv. Harosoy63) hypocotyls with 1 mM AgNO_3_ or water (control). Tissue samples were collected at 6, 12, 24, 48, or 72 hr after the treatment. Upon AgNO_3_ treatment, soybean hypocotyls displayed brown lesions at 12 h which became darker and bigger in the later time points. No such lesions were observed in the control hypocotyls at any time points (**Figure 5B**). RNA was isolated from these samples and expression profiles of 4 root-specific *P. sojae* induced *GmCHRs* were investigated in detail. Our qPCR results revealed that the expression of *GmCHR2A* was significantly higher at 6, 12, 24, and 48 h in AgNO_3_-treated soybean hypocotyls compared to the controls. Accumulation of *GmCHR14* and *GmCHR18* transcripts were significantly higher at 12, 24, 48, and 72 h post AgNO_3_ treatment compared to the control samples whereas increased accumulation of *GmCHR20* was only observed at 6 and 72 h in AgNO3-treated hypocotyls compared to the controls (**Figure 5C**).

### GmCHRs Localize in the Nucleus and Cytoplasm

**FIGURE 6:**
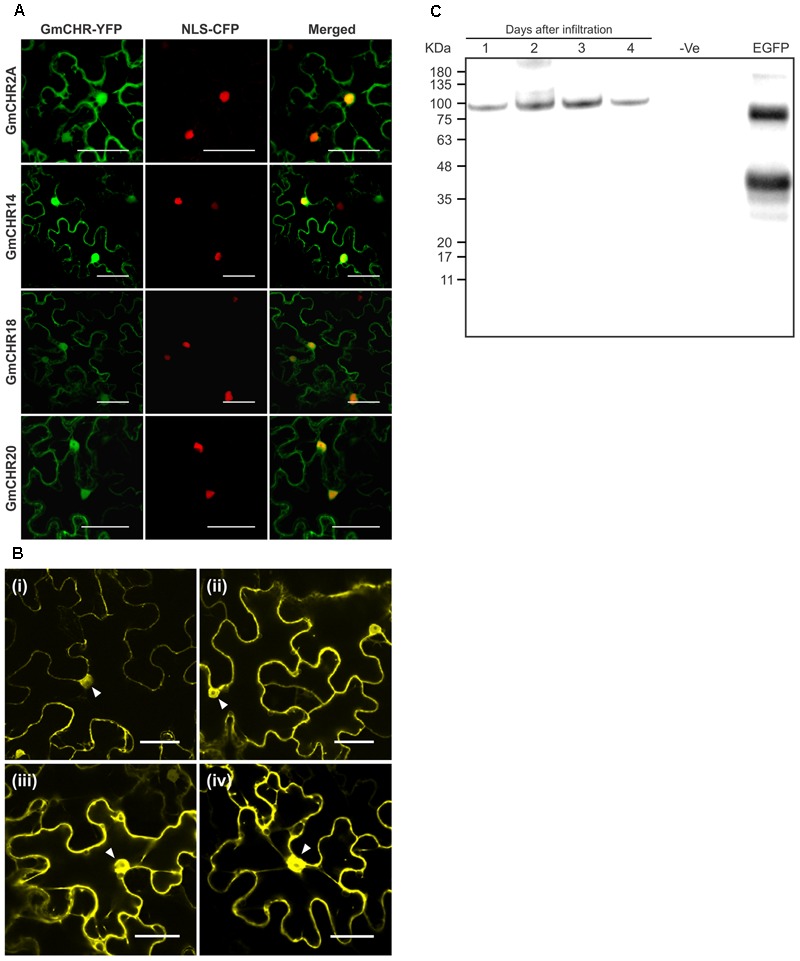
Subcellular localization of stress induced GmCHRs. **(A)** Four root-specific *GmCHR*s that show induced expression upon *P. sojae* infection were translationally fused with the YFP, and transiently expressed in *N. benthamiana* leaves by *A. tumefaciens*-mediated transformation followed by confocal microscopy. A nuclear localization signal fused with the CFP (NLS-CFP) was used to confirm nuclear localization. The scale bar indicates 50 μm. **(B)** Transient expression of GmCHR2A-YFP-YFP, in the epidermal cells of *N. benthamiana* as described in **(A)**. Pictures were taken as indicated (i) Day 1, (ii) Day 2, (iii) Day 3 and (iv) Day 4 post-infiltration. Triangles indicate the nucleus. **(C)** Western blot analysis of transiently expressed GmCHR2A-YFP-YFP in *N. benthamiana* leaves. Total proteins were separate on SDS-PAGE and transferred into a PVDF membrane, followed by Western blot analysis. The blot was incubated with anti-GFP primary antibody (1:7000 dilution) and HRP conjugated goat anti-mouse secondary antibody (1:5000 dilution), followed by chemiluminescence detection. Proteins extracted from an uninfiltrated *N. benthamiana* leaf sample was used as a negative control (–ve), and eGFP with hydrophobin was used as a positive control (∼37 kDa).

To determine the subcellular localization of the stress-induced *GmCHR* genes, each member was translationally fused with YFP, and the fusion protein was transiently expressed in leaf epidermal cells of *N. benthamiana* followed by confocal microscopy. All 4 GmCHRs (GmCHR2A, GmCHR14, GmCHR18 and GmCHR20) were found in 2 cellular compartments, cytoplasm and nucleus. Confirmation of the nuclear localization of GmCHRs was carried out by co-expressing GmCHR-YFP and nuclear localization signal (NLS) containing control (NLS-CFP), and observing the overlap between the NLS-CFP and GmCHR-YFP signals (**Figure 6A**). To demonstrate that the nuclear localization of GmCHRs are not due to passive diffusion into the nucleus, we made a translation fusions of two tandem YFP proteins with GmCHR2A (GmCHR2A-YFP-YFP) and determined its subcellular location. Despite of its larger molecular mass (88.39 kDa), the fusion protein GmCHR2A-YFP-YFP was found in both nucleus and cytoplasm (**Figure 6B**) suggesting that presence of GmCHR2A in the nucleus was not due to diffusion. Western blot analysis of transiently expressed GmCHR2A-YFP-YFP in *N. benthamiana* suggested that the fluorescence observed in the nucleus was due to the intact GmCHR2A-YFP-YFP and not by the cleaved YFP fragments as there was only a single band of estimated size and that the recombinant protein was not cleaved *in planta* by plant proteases (**Figure 6C**).

### QTLs and QTL Markers Linked to *P. sojae* Resistance Contain *GmCHR* Loci

To determine QTLs and QTL markers that are linked to *P. sojae* resistance, a survey of soybean database and literature search were conducted. A search in the ‘SoyBase and Soybean Breeder’s Toolbox’ from the year 2003 to 2016 identified 77 QTLs that are linked to *P. sojae* resistance in soybean (Supplementary Table S2). These 77 QTLs were extensively researched for *GmCHR* loci, parental lines and LOD scores. Four QTLs, Phytoph 10-3 (Han et al., 2008), Phytoph 14-3 (Lee et al., 2013), Phytoph 8-2 (Tucker et al., 2010) and Phytoph 15-5 (Wang et al., 2012) were found which contain *GmCHR* loci. Highlights of the QTLs included: (1) Phytoph 10-3 flanks *GmCHR2A* locus 2 megabase pairs and has an LOD score of 29.7; (2) Phytoph 14-3 and Phytoph 15-5 contain *GmCHR18* locus within the marker interval with lower LOD score; (3) Phytoph 8-2 contains *GmCHR20* locus that stretches over 31 megabase pairs with the LOD score of 4.5 (**Table 3**). These details suggest that Phytoph 10-3 containing *GmCHR2A* is involved in *P. sojae* resistance in soybean.

**Table 3:** Characteristics of QTLs linked to *P. sojae* resistance that contain *GmCHRs.*

QTL	Chr	QTL Marker Interval (physical map)	*GmCHR* and CHR location	Parents	Heritability	LOD	Reference
Phytoph 10-3	2	43,320,607..46,172,880	*GmCHR2A* 48,163,443 -48,164,792	Conrad OX760-6	n/a	29.77	Han et al., 2008
Phytoph 14-3	18	59,499,678.. 16,804,048	*GmCHR18* 56,611,421-56,613,070	OX20-8 PI 398841	0.77	3.4	Lee et al., 2013
Phytoph 15-5	18	53,866,536..57,968,533	*GmCHR18* 56,611,421-56,613,070	Conrad Sloan	n/a	8.4	Wang et al., 2012
Phytoph 8-2	20	35240575.. 3903416	*GmCHR20* 3,790,428-3,793,674	V71-370 PI407162	0.89	4.5	Tucker et al., 2010

*Chr, chromosome; LOD, logarithm of odds.*

An additional literature search was conducted for QTL markers linked to *P. sojae* resistance in soybean. This process identified over 500 QTL markers (Supplementary Table S3). The markers that share the same chromosome as *GmCHR* were separated, and exact locations of the QTL markers were noted. A total of 8 QTL markers were found to flank *GmCHR2A, GmCHR16A, GmCHR18* and *GmCHR20* loci (Han et al., 2008; Tucker et al., 2010; Wang et al., 2010). The details on the QTL marker are summarized in **Table 4**.

**Table 4:** Characteristics of QTL markers linked to *P. sojae* resistance that flank GmCHRs.

Gene	Chromosomal location	QTL marker	QTL marker location	Type of marker	Parents	Reference
*GmCHR2A*	Ch 02 48,163,443..48,164,792	Satt274	45,267,040..45,267,222	SSR	Conrad OX760-6	Han et al., 2008
*GmCHR16A*	Ch 16 37,672,779..37,675,776	Satt244	33,818,897..33,819,094	SSR	V71-370 PI407162	Tucker et al., 2010
		Q-16-0268535	33,793,393	SNP	MA	Huang et al., 2016
		BARC-042413-08254	35,175,092	SNP		
*GmCHR18*	Ch 18 56,611,421..56,613,070	BARC-039397-07314	56,889,971	SNP	Conrad Sloan	Wang et al., 2012
		BARCSOYSSR_18_1777	54,744,147..54,744,204	SSR		
		Satt472	53,866,606..53,866,716	SSR		
*GmCHR20*	Ch 20 3,790,324..3,793,674	Satt614	3,915,962..3,916,075	SSR	V71-370 PI407162	Tucker et al., 2010

*SSR, simple sequence repeats; SNP, single nucleotide polymorphism; MA, multiple accessions used.*

### *P. sojae* Resistant Soybean Cultivar Accumulates Higher Levels of Root-Specific *GmCHR*

**FIGURE 7:**
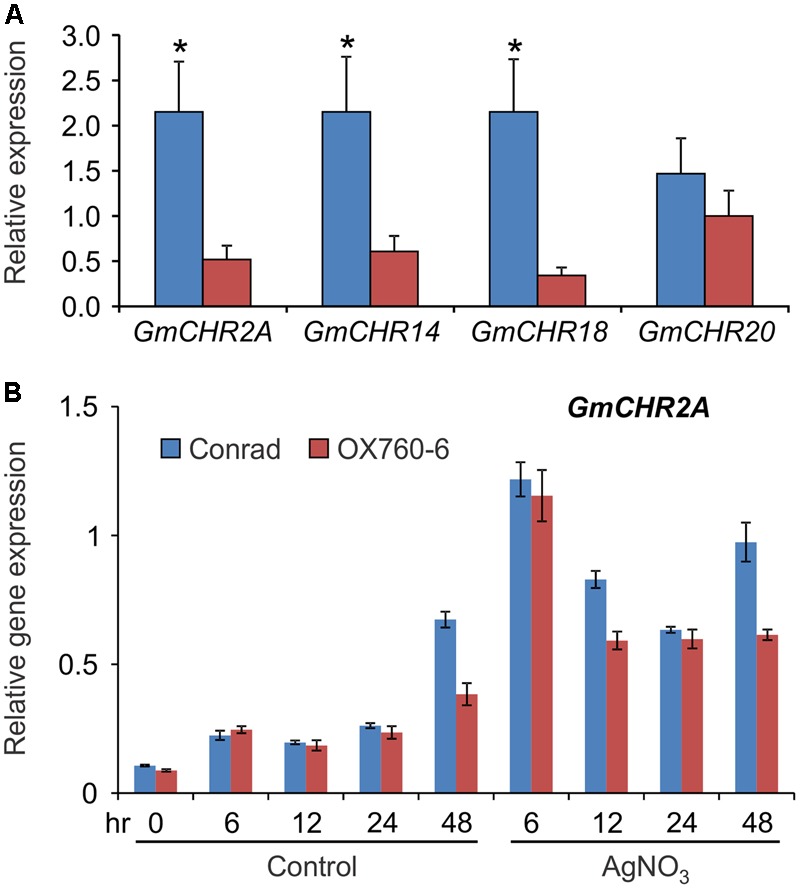
Accumulation of root-specific *GmCHR*s in Conrad and OX760-6. Total RNA (1 μg) was used to synthesize cDNA from **(A)** roots, and **(B)** untreated and AgNO3-treated etiolated hypocotyls of soybean cv. Conrad and OX760-6. Expression analysis was conducted by qPCR using gene-specific primers. Error bars indicates SEM of three technical replicates per biological replicates where 2 biological replicates for roots and 4 biological replicates for etiolated hypocotyls were used. *CONS4* was used as a reference gene. The asterisks (^∗^) denotes significant difference in expression as determined by Student’s *t*-test (*p* < 0.05).

Since several QTL markers and QTLs linked to *P. sojae* resistance contained *GmCHR* gene family members, we evaluated the parental cultivars of QTL Phytoph 10-3 for root-specific *GmCHR* gene expression. Root tissues of 2-week old seedlings of soybean cv. Conrad and OX760-6 were used for gene expression analysis. As shown in **Figure 7A**, expression of *GmCHR2A, GmCHR14* and *GmCHR18* were significantly higher in roots of Conrad (*P. sojae* resistant cultivar) compared to the OX760-6 (*P. sojae* susceptible cultivar). No difference in the expression of *GmCHR20* was observed between Conrad and OX760-6. When the etiolated hypocotyls of Conrad and OX760-6 were treated with AgNO_3_, and expression of root-specific *GmCHR*s were monitored, only the expression of *GmCHR2A* was induced in response to AgNO_3_ treatment and its transcript accumulation was higher in Conrad compared to OX760-6 at later time points (**Figure 7B**). No difference in the level of other root-specific *GmCHRs* were observed in Conrad and OX760-6 both in control and AgNO_3-_treated samples. Furthermore, expression of *GmCHR14* and *GmCHR18* were not induced in response to AgNO_3_ treatment (not shown).

## Discussion

The legume-specific enzyme CHR together with CHS, converts 1 molecule of *p*-coumaroyl-CoA and 3 molecules of malonyl-CoA to isoliquiritigenin, the building block of two core isoflavone aglycones, glycitein and daidzein, where daidzein serves as a precursor for the production of phytoalexin glyceollins in soybean. Many studies have reported the expression patterns of genes during infection and the heritability of resistance, however, little is known about the importance of *CHR*, the first key enzyme, which directs the flux to the production of phytoalexin glyceollins in soybean. Here we report the genome-wide identification of *GmCHRs* in soybean, investigate their subcellular location, and tissue-specific and pathogen induced gene expression. Our results demonstrate that the root-specific *GmCHRs* are induced upon pathogen infection, and are located near QTLs and QTL markers linked to *P. sojae* resistance.

Many CHR-like enzymes have been reported in a variety of leguminous plants (Ballance and Dixon, 1995; Goormachtig et al., 1999; Shimada et al., 2006; He et al., 2011; Hayashi et al., 2013). A recent study identified only 2 *CHRs* (*GmCHR2A* and *GmCHR14*) in soybean where only one sequence from GenBank (accession EU921437) was used as the query to search the soybean genome database (Chu et al., 2014). Here we identified 14 *CHR* gene family members in soybean. Our approach is more robust and provides confidence as it first identified all the 67 *GmAKR*s based on the current database annotation and other search tools, and then segregated the *GmCHRs* gene family members using their phylogenetic relationship with known CHRs (**Figure 2**). We also confirmed that the 4 *GmCHR*s previously identified (Subramanian et al., 2006) are also included in our list. Soybean is a paleopolyploid with a genome size of approximately 1 gigabase pairs that has undergone at least two whole genome duplications (Schmutz et al., 2010). As a result of the genome duplications, nearly 75% of soybean genes are present in multiple copies. The large number of *GmCHRs* could be the result of whole genome duplication events in soybean. Phylogenetic analysis of GmCHRs illustrated that most GmCHRs cluster in pairs, further supporting ancient genome duplication events (**Figure 2**).

Out of 14 putative GmCHRs, 11 were found to contain conserved critical residues (**Figure 3**). Since CHRs are a part of the AKR family, these enzymes must contain the catalytic sites (Bomati et al., 2005). The 3 GmCHRs: GmCHR2B, GmCHR9B and GmCHR16C lack one or more catalytic site residues, therefore, were eliminated from our study. However, it is possible that they may possess weak enzymatic activity or may be evolving new catalytic features.

*GmCHR*s displayed tissue-specific gene expression. The majority of the *GmCHRs* were either expressed in the seedlings, roots or dry seeds (**Figure 4**). Previously, it was found that *CHR* in soybean were moderately expressed in the flowers and weakly expressed in leaves, stems, roots, endosperms and embryos (Liu, 2009). Differential expression of *CHR*s have also been studied in other plant species such as *Astragalus membranaceus* (Xu et al., 2012) and *P. montana* var. lobata (He et al., 2011). It was found that *CHRs* from *A. membranaceus* and *P. montana* var. lobata were highly expressed in roots and stem, respectively. Out of 11 *GmCHRs* identified, *GmCHR2A, GmCHR14, GmCHR12, GmCHR18* and *GmCHR20* transcript accumulation was much higher in root tissue compared to other tissues under study. Studies have shown that *CHS7, CHS8* (Yi et al., 2010), *IFS1, IFS2* (Dhaubhadel et al., 2003) and *CHI* (Dastmalchi and Dhaubhadel, 2015) are also expressed in the roots which infers that they possibly assist in the role of root-specific phytoalexins production.

To evaluate if *GmCHR* family members respond differently upon pathogen infection, their expression levels were studied by RT-PCR at various time points after *P. sojae* infection. Interestingly, the expression levels of only 4 root-specific *GmCHRs, GmCHR2A, GmCHR14, GmCHR18* and GmCHR*20*, were induced upon infection suggesting that they have a role in defense against *P. sojae* infection (**Figure 5A**). Upon infection, *P. sojae* releases elicitors which stimulate the plant defense response (Jones and Dangl, 2006). As a result, the plant induces the expression of resistance and defense related genes to counteract infection. Studies have shown the upregulation or induction of *CHR*s at the infection site during *Fusarium* attack in soybeans (Iqbal et al., 2005), cadmium treatment in *Medicago truncatula* (Aloui et al., 2012), and *Colletotrichum falcatum* infection in sugarcane (Selvaraj et al., 2014). Several studies have used the AgNO_3_ treatment to mimic pathogen infection and induce phytoalexin production in soybeans (Moy et al., 2004; Kubeš et al., 2014). The mechanism of this “elicitor effect” has not been identified yet. The quantitative analysis of root-specific *GmCHRs* in response to AgNO_3_ demonstrated a significant increase in transcript accumulation of *GmCHR2A, GmCHR14* and *GmCHR18* (**Figure 5C**). These root-specific *GmCHRs* respond to the AgNO_3_ treatment as early as 12 h which coincides with findings from (Alkharouf et al., 2006). Changes in gene expression within roots upon *Heterodera glycines* (the soybean cyst nematode) attack was investigated using a 6000 gene microarray (Alkharouf et al., 2006). It was found that *CHR* (Genbank BM108162) was induced as soon as 6 and 12 h upon infection.

All members of the GmCHR family displayed nuclear and cytoplasmic localization in *N. benthamiana* leaf epidermal cells (**Figure 6**). These findings are consistent with the localization of GmCHS which works together with GmCHR to produce isoliquiritigenin. Evidence has shown that other enzymes involved in the isoflavonoid biosynthesis such as GmCHI (Dastmalchi and Dhaubhadel, 2015), glycosyltransferase (UGT73F2) and malonyltransferase (GmMT7) (Dhaubhadel et al., 2008) are also localized to the nucleus and the cytoplasm. Similarly, nuclear localization of flavonoid enzymes have also been reported in *Arabidopsis* (Saslowsky et al., 2005). Flavanols and flavonols have been found in the nuclei of some tree species (Feucht et al., 2012) such as conifers *Taxus baccata* L., *Tsuga canadensis* L. (Feucht et al., 2014), however, a simple diffusion process was speculated for the presence of these metabolites in the nucleus. Since isoflavonoid biosynthesis requires multiple cytochrome P450 monooxygenases that are localized in the endoplasmic reticulum, synthesis of these metabolites in the nucleus can be completely ruled out. This could imply that nuclear localized GmCHRs might have an alternate function other than their catalytic role in the isoflavonoid biosynthesis. It is possible that the nuclear localized isoflavonoid enzymes may function as moonlighting proteins with two or more different functions at different locations within the cell (Copley, 2012; Jeffery, 2014).

Several studies have suggested that *Rps* genes (Dorrance and Schmitthenner, 2000; Sandhu et al., 2005), isoflavonoid biosynthetic genes (Subramanian et al., 2005; Graham et al., 2007), and genes involved in suberin production (Ranathunge et al., 2008) contribute to *P. sojae* resistance in soybean. Furthermore, molecular markers linked to *Rps* genes or QTLs and QTL markers linked to *P. sojae* resistance have been identified in soybean. However, little has been reported linking the candidate genes with the QTLs and the phenotype. Here, we have identified a total of 8 QTL markers and 4 QTLs which flank or are approximate to *GmCHR2A, GmCHR18* and *GmCHR20*. Among the QTLs, Phytoph 14-3 covers most of the chromosome 18 (**Table 3**). Even though QTL regions can generally span several megabase pairs, and can contain several hundreds to thousands of genes (Dupuis and Siegmund, 1999), further fine-mapping experiments are required to pinpoint the spanning regions (Touzet et al., 1995; Holtan and Hake, 2003). Statistical association or validation studies can confirm the co-segregation of genes with a QTL. Based on these parameters, QTL Phytoph 10-3 was selected as the most reliable QTL found in this study as it is confined to a specific region on the chromosome and contains the highest LOD score. Even though the expression of root-specific *GmCHRs, GmCHR2A, GmCHR14* and *GmCHR18* were significantly higher in the roots of the parental cultivars Conrad (resistant cultivar) as compared to OX760-6 (susceptible cultivar) used in the QTL study by Han et al. (2008), only expression of *GmCHR2A* was induced upon stress and was higher in the cultivar Conrad as compared to OX760-6 (**Figure 7B**). This result together with the expression of root-specific *GmCHRs* (**Figure 4**), suggests that in addition to GmCHRs, there are other yet unidentified factors that play an important role in partial resistance in soybean. Identification of each component is crucial to understand the resistance mechanism so that genes encoding those factors could be stacked together into a single cultivar for resistance breeding.

## Author Contributions

SD conceived and designed the experiments. CS and JY performed the experiments and analyzed the data. SD and CS wrote the article; all the authors read and commented on the article.

## Conflict of Interest Statement

The authors declare that the research was conducted in the absence of any commercial or financial relationships that could be construed as a potential conflict of interest.

## Footnotes

1 www.globalsoybeanproduction.com

2 soycanada.ca/statistics/at-a-glance/

3 http://compbio.dfci.harvard.edu/tgi/

4 http://phytozome.jgi.doe.gov/pz/portal.html

5 http://www.ebi.ac.uk/Tools/msa/clustalo/

6 http://www.ch.embnet.org/software/BOX_form.html

7 http://soybase.org/

8 http://www.ncbi.nlm.nih.gov/geo/query/acc.cgi?acc=GSE29163

9 http://www.ncbi.nlm.nih.gov/geo/query/acc.cgi?acc=GSE29163

## Supplementary Material

FIGURE S1 Set-up of AgNO_3_ stress treatment on soybean hypocotyl.
