# Supplementary figures and images for: Genome-Wide Identification of Chalcone Reductase Gene Family in Soybean: Insight into Root-Specific GmCHRs and Phytophthora sojae Resistance
Source: Front Plant Sci. 2017 Dec 7;8:2073. doi: 10.3389/fpls.2017.02073 (PMC5725808; doi:10.3389/fpls.2017.02073)

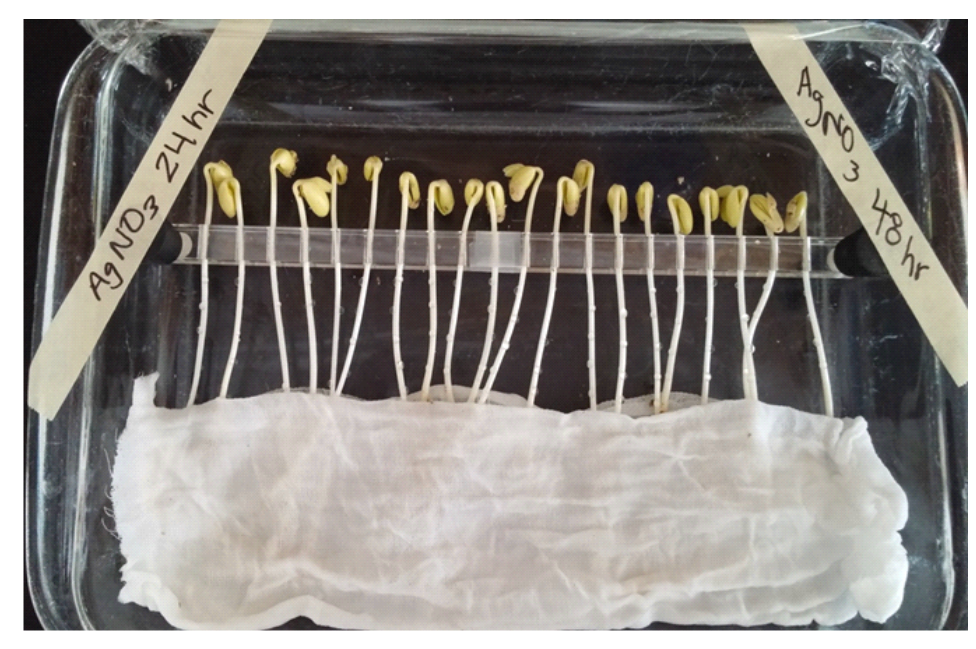

Supplement: FIGURE S1 — Set-up of AgNO3 stress treatment on soybean hypocotyl. [file Image_1.TIF]
