# Supplementary material for: Genome-Wide Identification of Chalcone Reductase Gene Family in Soybean: Insight into Root-Specific GmCHRs and Phytophthora sojae Resistance
Source: Front Plant Sci. 2017 Dec 7;8:2073. doi: 10.3389/fpls.2017.02073 (PMC5725808; doi:10.3389/fpls.2017.02073)
Supplement: Supplementary file 2 [file Table_1.DOCX]

**Table S1. List of primers used for cloning and qPCR of *GmCHRs*.**

| Gene | Primer name | Sequence (5’-3’) | Amplicon size | Purpose |
| --- | --- | --- | --- | --- |
| *GmCHR2A* | GmCHR2AF | GGGGACAAGTTTGTACAAAAAAGCAGGCTTCGGTTATAGTATTTGTAATACAATGGCGGCT | 967 | Subcellular localization and RT-PCR |
|  | GmCHR2AR | GGGGACCACTTTGTACAAGAAAGCTGGGTCTATTTCATCATCCCAGAGATCAGTGAC |  |  |
| *GmCHR9A* | GmCHR9AF | GGA AGC AAA GAA AAT CCC AGA AGT G | 932 |  |
|  | GmCHR9AR | TCA GGG GTC TCC ATC CCA AAG |  |  |
| *GmCHR9D* | GmCHR9DF | ATG AGG AGC AAT CAT GTG CGT TTG | 922 |  |
|  | GmCHR9DR | CGA TTG TTC TGT AGG GAC TTG TGG |  |  |
| *GmCHR9C* | GmCHR9CF | GGGGACAAGTTTGTACAAAAAAGCAGGCTTCATGGAACCAAAGGCAATCCAA | 945 |  |
|  | GmCHR9CR | GGGGACCACTTTGTACAAGAAAGCTGGGTCATCCCAAAAATCTTGTGGAGTTTTGT |  |  |
| *GmCHR12* | GmCHR12F | ATG AGG AGC AAT CAT GTG CG | 945 |  |
|  | GmCHR12R | AAT TTC ATC ATC CCA AAG TTC TTG G |  |  |
| *GmCHR14* | GmCHR14F | GGGGACAAGTTTGTACAAAAAAGCAGGCTTCCTTGTCAACCCTTTGAGAGTTAGAATG | 1002 |  |
|  | GmCHR14R | GGGGACCACTTT GTACAAGAAAGCTGGGTCTATTTGATCATCCCAGAGATCAGC |  |  |
| *GmCHR15* | GmCHR15F | ATG TCT TCA TCA AAC ATC CCT CAT | 945 |  |
|  | GmCHR15R | ATT TTC TCC ATC CCA AAG GTC A |  |  |
| *GmCHR16A* | GmCHR16AF | GGGGACAAGTTTGTACAAAAAAGCAGGCTTCTCAGCAGACCCAAAAGAAGATATG | 981 |  |
|  | GmCHR16AR | GGGGACCACTTTGTACAAGAAAGCTGGGTCATCCCAAAGCTCTTCCAAGG |  |  |
| *GmCHR16B* | GmCHR16BF | GGGGACAAGTTTGTACAAAAAAGCAGGCTTCAGTTAGAAACAAGGAGAAAGAAAGAAGATG | 993 |  |
|  | GmCHR16BR | GGGGACCACTTTGTACAAGAAAGCTGGGTCAACGTCTCCATCCCAAAGTTC |  |  |
| *GmCHR18* | GmCHR18F | GGGGACAAGTTTGTACAAAAAAGCAGGCTTCATGGCTGCCACCACCTTAGT | 945 |  |
|  | GmCHR18R | GGGGACCACTTTGTACAAGAAAGCTGGGTCTTCTTCATCCCATAGATCATCAAGAAGAGG |  |  |
| *GmCHR20* | GmCHR20F | GGGGACAAGTTTGTACAAAAAAGCAGGCTTCGACACATCGCAACATCGTATCATG | 984 |  |
|  | GmCHR20R | GGGGACCACTTTGTACAAGAAAGCTGGGTCAGTTTCCCCATCAAATAGATCAGG |  |  |
| *GmCHR2A* | qGmCHR2AF | CGGGGAGGTGCTTCTTGGTTATAG | 150 | qPCR |
|  | qGmCHR2AR | CTCCTTAGTGTCTTTCTTGCA |  |  |
| *GmCHR14* | qGmCHR14F | CCACCTACCTCCAATTGCTGAC | 167 |  |
|  | qGMCHR14R | GGGATTTCAATAGCAGCAGCC |  |  |
| *GmCHR18* | qGmCHR18F | ATCCCACCTACCCACATCGT | 175 |  |
|  | qGmCHR18R | GGAGGAGGGGAGAGTGACTT |  |  |
| *GmCHR20* | qGmCHR20F | CACTCGTATCTGTGACACCGTGT | 201 |  |
