# Supplementary material for: Genome-Wide Identification of Chalcone Reductase Gene Family in Soybean: Insight into Root-Specific GmCHRs and Phytophthora sojae Resistance
Source: Front Plant Sci. 2017 Dec 7;8:2073. doi: 10.3389/fpls.2017.02073 (PMC5725808; doi:10.3389/fpls.2017.02073)
Supplement: Supplementary file 3 [file Table_2.DOCX]

**Table S2 QTLs linked with *P. sojae* resistance in soybean (from 2003-2016)**

| QTL | Chromosome | Parents | Reference |
| --- | --- | --- | --- |
| [Phytoph 1-1](http://www.soybase.org/sbt/search/search_results.php?category=QTLName&search_term=Phytoph%201-1) | 13 | Parent 1: Conrad  Parent 2: Harosoy | ([Tucker et al., 2010](#_ENREF_96)) |
| [Phytoph 1-2](http://www.soybase.org/sbt/search/search_results.php?category=QTLName&search_term=Phytoph%201-2) | 2 | Parent 1: Conrad  Parent 2: Harosoy | ([Tucker et al., 2010](#_ENREF_96)) |
| [Phytoph 10-1](http://www.soybase.org/sbt/search/search_results.php?category=QTLName&search_term=Phytoph%2010-1) | 13 | Parent 1: Conrad  Parent 2: OX760-6-1 | ([Han et al., 2008](#_ENREF_45)) |
| [Phytoph 10-2](http://www.soybase.org/sbt/search/search_results.php?category=QTLName&search_term=Phytoph%2010-2) | 13 | Parent 1: Conrad  Parent 2: OX760-6-1 | ([Han et al., 2008](#_ENREF_45)) |
| [Phytoph 10-3](http://www.soybase.org/sbt/search/search_results.php?category=QTLName&search_term=Phytoph%2010-3) | 2 | Parent 1: Conrad  Parent 2:OX760-6-1 | ([Han et al., 2008](#_ENREF_45)) |
| Phytoph 11-19 | 13 | Parent 1: Conrad  Parent 2: Sloan | ([Wang et al., 2012](#_ENREF_98)A) |
| [Phytoph 11-2](http://www.soybase.org/sbt/search/search_results.php?category=QTLName&search_term=Phytoph%2011-2) | 8 | Parent 1: Conrad  Parent 2: Sloan | ([Wang et al., 2012](#_ENREF_98)A) |
| [Phytoph 11-20](http://www.soybase.org/sbt/search/search_results.php?category=QTLName&search_term=Phytoph%2011-20) | 17 | Parent 1: Conrad  Parent 2: Sloan | ([Wang et al., 2012](#_ENREF_98)A) |
| [Phytoph 11-21](http://www.soybase.org/sbt/search/search_results.php?category=QTLName&search_term=Phytoph%2011-21) | 13 | Parent 1: Conrad  Parent 2: Sloan | ([Wang et al., 2012](#_ENREF_98)A) |
| [Phytoph 11-22](http://www.soybase.org/sbt/search/search_results.php?category=QTLName&search_term=Phytoph%2011-22) | 14 | Parent 1: Conrad  Parent 2: Sloan | ([Wang et al., 2012](#_ENREF_98)A) |
| [Phytoph 12-1](http://www.soybase.org/sbt/search/search_results.php?category=QTLName&search_term=Phytoph%2012-1) | 13 | Parent 1: S99-2281  Parent 2:PI 408105A | ([Nguyen et al., 2012](#_ENREF_73)) |
| [Phytoph 12-2](http://www.soybase.org/sbt/search/search_results.php?category=QTLName&search_term=Phytoph%2012-2) | 17 | Parent 1: S99-2281  Parent 2: PI 408105A | ([Nguyen et al., 2012](#_ENREF_73)) |
| [Phytoph 13-1](http://www.soybase.org/sbt/search/search_results.php?category=QTLName&search_term=Phytoph%2013-1) | 19 | Parent 1: Conrad  Parent 2: Sloan | ([Wang et al., 2012](#_ENREF_100)B) |
| [Phytoph 13-2](http://www.soybase.org/sbt/search/search_results.php?category=QTLName&search_term=Phytoph%2013-2) | 19 | Parent 1: Conrad  Parent 2: Sloan | ([Wang et al., 2012](#_ENREF_100)B) |
| [Phytoph 13-3](http://www.soybase.org/sbt/search/search_results.php?category=QTLName&search_term=Phytoph%2013-3) | 1 | Parent 1: Conrad  Parent 2: Sloan | ([Wang et al., 2012](#_ENREF_100)B) |
| [Phytoph 13-4](http://www.soybase.org/sbt/search/search_results.php?category=QTLName&search_term=Phytoph%2013-4) | 18 | Parent 1: Conrad  Parent 2: Sloan | ([Wang et al., 2012](#_ENREF_100)B) |
| [Phytoph 13-5](http://www.soybase.org/sbt/search/search_results.php?category=QTLName&search_term=Phytoph%2013-5) | 18 | Parent 1: Conrad  Parent 2: Sloan | ([Wang et al., 2012](#_ENREF_100)B) |
| [Phytoph 14-1](http://www.soybase.org/sbt/search/search_results.php?category=QTLName&search_term=Phytoph%2014-1) | 1 | Parent 1: OX20-8  Parent 2: PI 398841 | ([Lee et al., 2013](#_ENREF_64)) |
| [Phytoph 14-10](http://www.soybase.org/sbt/search/search_results.php?category=QTLName&search_term=Phytoph%2014-10) | 20 | Parent 1: OX20-8  Parent 2: PI 398841 | ([Lee et al., 2013](#_ENREF_64)) |
| [Phytoph 14-2](http://www.soybase.org/sbt/search/search_results.php?category=QTLName&search_term=Phytoph%2014-2) | 13 | Parent 1: OX20-8  Parent 2: PI 398841 | ([Lee et al., 2013](#_ENREF_64)) |
| [Phytoph 14-3](http://www.soybase.org/sbt/search/search_results.php?category=QTLName&search_term=Phytoph%2014-3) | 18 | Parent 1: OX20-8  Parent 2: PI 398841 | ([Lee et al., 2013](#_ENREF_64)) |
| [Phytoph 14-4](http://www.soybase.org/sbt/search/search_results.php?category=QTLName&search_term=Phytoph%2014-4) | 2 | Parent 1: OX20-8  Parent 2: PI 398841 | ([Lee et al., 2013](#_ENREF_64)) |
| [Phytoph 14-5](http://www.soybase.org/sbt/search/search_results.php?category=QTLName&search_term=Phytoph%2014-5) | 3 | Parent 1: OX20-8  Parent 2: PI 398841 | ([Lee et al., 2013](#_ENREF_64)) |
| [Phytoph 14-6](http://www.soybase.org/sbt/search/search_results.php?category=QTLName&search_term=Phytoph%2014-6) | 4 | Parent 1: OX20-8  Parent 2: PI 398841 | ([Lee et al., 2013](#_ENREF_64)) |
| [Phytoph 14-7](http://www.soybase.org/sbt/search/search_results.php?category=QTLName&search_term=Phytoph%2014-7) | 4 | Parent 1: OX20-8  Parent 2: PI 398841 | ([Lee et al., 2013](#_ENREF_64)) |
| [Phytoph 14-8](http://www.soybase.org/sbt/search/search_results.php?category=QTLName&search_term=Phytoph%2014-8) | 7 | Parent 1: OX20-8  Parent 2: PI 398841 | ([Lee et al., 2013](#_ENREF_64)) |
| [Phytoph 14-9](http://www.soybase.org/sbt/search/search_results.php?category=QTLName&search_term=Phytoph%2014-9) | 15 | Parent 1: OX20-8  Parent 2: PI 398841 | ([Lee et al., 2013](#_ENREF_64)) |
| [Phytoph 2-1](http://www.soybase.org/sbt/search/search_results.php?category=QTLName&search_term=Phytoph%202-1) | 13 | Parent 1: Conrad  Parent 2: Sloan | ([Burnham et al., 2003](#_ENREF_11)) |
| [Phytoph 2-2](http://www.soybase.org/sbt/search/search_results.php?category=QTLName&search_term=Phytoph%202-2) | 2 | Parent 1: Conrad  Parent 2: Sloan | ([Burnham et al., 2003](#_ENREF_11)) |
| [Phytoph 3-1](http://www.soybase.org/sbt/search/search_results.php?category=QTLName&search_term=Phytoph%203-1) | 13 | Parent 1: Conrad  Parent 2: Harosoy | ([Burnham et al., 2003](#_ENREF_11)) |
| [Phytoph 3-2](http://www.soybase.org/sbt/search/search_results.php?category=QTLName&search_term=Phytoph%203-2) | 2 | Parent 1: Conrad  Parent 2: Harosoy | ([Burnham et al., 2003](#_ENREF_11)) |
| [Phytoph 4-1](http://www.soybase.org/sbt/search/search_results.php?category=QTLName&search_term=Phytoph%204-1) | 13 | Parent 1: Conrad  Parent 2: Williams | ([Burnham et al., 2003](#_ENREF_11)) |
| [Phytoph 4-2](http://www.soybase.org/sbt/search/search_results.php?category=QTLName&search_term=Phytoph%204-2) | 2 | Parent 1: Conrad  Parent 2: Williams | ([Burnham et al., 2003](#_ENREF_11)) |
| [Phytoph 5-1](http://www.soybase.org/sbt/search/search_results.php?category=QTLName&search_term=Phytoph%205-1) | 6 | Parent 1: Su88-M21(S)  Parent 2: Xinyixiaoheidou (X) | ([Wu et al., 2011](#_ENREF_104)) |
| [Phytoph 5-2](http://www.soybase.org/sbt/search/search_results.php?category=QTLName&search_term=Phytoph%205-2) | 15 | Parent 1: Su88-M21(S)  Parent 2: Xinyixiaoheidou (X) | ([Wu et al., 2011](#_ENREF_104)) |
| [Phytoph 5-3](http://www.soybase.org/sbt/search/search_results.php?category=QTLName&search_term=Phytoph%205-3) | 10 | Parent 1: Su88-M21(S)  Parent 2: Xinyixiaoheidou (X) | ([Wu et al., 2011](#_ENREF_104)) |
| [Phytoph 6-1](http://www.soybase.org/sbt/search/search_results.php?category=QTLName&search_term=Phytoph%206-1) | 13 | Parent 1: Conrad  Parent 2: Hefeng 25 | ([Li et al., 2010](#_ENREF_65)) |
| [Phytoph 6-2](http://www.soybase.org/sbt/search/search_results.php?category=QTLName&search_term=Phytoph%206-2) | 2 | Parent 1: Conrad  Parent 2: Hefeng 25 | ([Li et al., 2010](#_ENREF_65)) |
| [Phytoph 6-3](http://www.soybase.org/sbt/search/search_results.php?category=QTLName&search_term=Phytoph%206-3) | 2 | Parent 1: Conrad  Parent 2: Hefeng 25 | ([Li et al., 2010](#_ENREF_65)) |
| [Phytoph 6-4](http://www.soybase.org/sbt/search/search_results.php?category=QTLName&search_term=Phytoph%206-4) | 8 | Parent 1: Conrad  Parent 2: Hefeng 25 | ([Li et al., 2010](#_ENREF_65)) |
| [Phytoph 6-5](http://www.soybase.org/sbt/search/search_results.php?category=QTLName&search_term=Phytoph%206-5) | 11 | Parent 1: Conrad  Parent 2: Hefeng 25 | ([Li et al., 2010](#_ENREF_65)) |
| [Phytoph 6-6](http://www.soybase.org/sbt/search/search_results.php?category=QTLName&search_term=Phytoph%206-6) | 6 | Parent 1: Conrad  Parent 2: Hefeng 25 | ([Li et al., 2010](#_ENREF_65)) |
| [Phytoph 6-7](http://www.soybase.org/sbt/search/search_results.php?category=QTLName&search_term=Phytoph%206-7) | 6 | Parent 1: Conrad  Parent 2: Hefeng 25 | ([Li et al., 2010](#_ENREF_65)) |
| [Phytoph 6-8](http://www.soybase.org/sbt/search/search_results.php?category=QTLName&search_term=Phytoph%206-8) | 6 | Parent 1: Conrad  Parent 2: Hefeng 25 | ([Li et al., 2010](#_ENREF_65)) |
| [Phytoph 7-1](http://www.soybase.org/sbt/search/search_results.php?category=QTLName&search_term=Phytoph%207-1) | 16 | Parent 1:Conrad  Parent 2:OX760-6-1 | ([Weng et al., 2007](#_ENREF_102)) |
| [Phytoph 8-1](http://www.soybase.org/sbt/search/search_results.php?category=QTLName&search_term=Phytoph%208-1) | 16 | Parent 1: V71-370  Parent 2: PI407162 | ([Tucker et al., 2010](#_ENREF_96)) |
| [Phytoph 8-2](http://www.soybase.org/sbt/search/search_results.php?category=QTLName&search_term=Phytoph%208-2) | 20 | Parent 1: V71-370  Parent 2: PI407162 | ([Tucker et al., 2010](#_ENREF_96)) |
| [Phytoph 8-3](http://www.soybase.org/sbt/search/search_results.php?category=QTLName&search_term=Phytoph%208-3) | 18 | Parent 1: V71-370  Parent 2: PI407162 | ([Tucker et al., 2010](#_ENREF_96)) |
| [Phytoph 8-4](http://www.soybase.org/sbt/search/search_results.php?category=QTLName&search_term=Phytoph%208-4) | 13 | Parent 1:V71-370  Parent 2:PI407162 | ([Tucker et al., 2010](#_ENREF_96)) |
| [Phytoph 9-1](http://www.soybase.org/sbt/search/search_results.php?category=QTLName&search_term=Phytoph%209-1) | 12 | Parent 1: Conrad  Parent 2: Sloan | ([Wang et al., 2010](#_ENREF_99)) |
| [Phytoph 9-2](http://www.soybase.org/sbt/search/search_results.php?category=QTLName&search_term=Phytoph%209-2) | 13 | Parent 1: Conrad  Parent 2: Sloan | ([Wang et al., 2010](#_ENREF_99)) |
| [Phytoph 9-3](http://www.soybase.org/sbt/search/search_results.php?category=QTLName&search_term=Phytoph%209-3) | 13 | Parent 1: Conrad  Parent 2: Sloan | ([Wang et al., 2010](#_ENREF_99)) |
| [Phytoph 9-4](http://www.soybase.org/sbt/search/search_results.php?category=QTLName&search_term=Phytoph%209-4) | 14 | Parent 1: Conrad  Parent 2: Sloan | ([Wang et al., 2010](#_ENREF_99)) |
| [Phytoph 9-5](http://www.soybase.org/sbt/search/search_results.php?category=QTLName&search_term=Phytoph%209-5) | 17 | Parent 1: Conrad  Parent 2: Sloan | ([Wang et al., 2010](#_ENREF_99)) |
| [Phytoph 9-6](http://www.soybase.org/sbt/search/search_results.php?category=QTLName&search_term=Phytoph%209-6) | 19 | Parent 1: Conrad  Parent 2: Sloan | ([Wang et al., 2010](#_ENREF_99)) |
| Phytoph 11-1 | 8 | Parent 1: Conrad  Parent 2: Sloan | (Wang et al., 2012A) |
| Phytoph 11-3 | 18 | Parent 1: Conrad  Parent 2: Sloan | (Wang et al., 2012A) |
| [Phytoph 11-4](https://www.soybase.org/sbt/search/search_results.php?category=QTLName&search_term=Phytoph+11-4) | 18 | Parent 1: Conrad  Parent 2: Sloan | (Wang et al., 2012A) |
| [Phytoph 11-5](https://www.soybase.org/sbt/search/search_results.php?category=QTLName&search_term=Phytoph+11-5) | 18 | Parent 1: Conrad  Parent 2: Sloan | (Wang et al., 2012A) |
| [Phytoph 11-6](https://www.soybase.org/sbt/search/search_results.php?category=QTLName&search_term=Phytoph+11-6) | 18 | Parent 1: Conrad  Parent 2: Sloan | (Wang et al., 2012A) |
| [Phytoph 11-7](https://www.soybase.org/sbt/search/search_results.php?category=QTLName&search_term=Phytoph+11-7) | 19 | Parent 1: Conrad  Parent 2: Sloan | (Wang et al., 2012A) |
| [Phytoph 11-8](https://www.soybase.org/sbt/search/search_results.php?category=QTLName&search_term=Phytoph+11-8) | 19 | Parent 1: Conrad  Parent 2: Sloan | (Wang et al., 2012A) |
| [Phytoph 11-9](https://www.soybase.org/sbt/search/search_results.php?category=QTLName&search_term=Phytoph+11-9) | 19 | Parent 1: Conrad  Parent 2: Sloan | (Wang et al., 2012A) |
| [Phytoph 11-10](https://www.soybase.org/sbt/search/search_results.php?category=QTLName&search_term=Phytoph+11-10) | 19 | Parent 1: Conrad  Parent 2: Sloan | (Wang et al., 2012A) |
| [Phytoph 11-11](https://www.soybase.org/sbt/search/search_results.php?category=QTLName&search_term=Phytoph+11-11) | 19 | Parent 1: Conrad  Parent 2: Sloan | (Wang et al., 2012A) |
| [Phytoph 11-12](https://www.soybase.org/sbt/search/search_results.php?category=QTLName&search_term=Phytoph+11-12) | 19 | Parent 1: Conrad  Parent 2: Sloan | (Wang et al., 2012A) |
| [Phytoph 11-13](https://www.soybase.org/sbt/search/search_results.php?category=QTLName&search_term=Phytoph+11-13) | 19 | Parent 1: Conrad  Parent 2: Sloan | (Wang et al., 2012A) |
| [Phytoph 11-14](https://www.soybase.org/sbt/search/search_results.php?category=QTLName&search_term=Phytoph+11-14) | 19 | Parent 1: Conrad  Parent 2: Sloan | (Wang et al., 2012A) |
| [Phytoph 11-15](https://www.soybase.org/sbt/search/search_results.php?category=QTLName&search_term=Phytoph+11-15) | 19 | Parent 1: Conrad  Parent 2: Sloan | (Wang et al., 2012A) |
| [Phytoph 11-16](https://www.soybase.org/sbt/search/search_results.php?category=QTLName&search_term=Phytoph+11-16) | 19 | Parent 1: Conrad  Parent 2: Sloan | (Wang et al., 2012A) |
| [Phytoph 11-17](https://www.soybase.org/sbt/search/search_results.php?category=QTLName&search_term=Phytoph+11-17) | 12 | Parent 1: Conrad  Parent 2: Sloan | (Wang et al., 2012A) |
| [Phytoph 11-18](https://www.soybase.org/sbt/search/search_results.php?category=QTLName&search_term=Phytoph+11-18) | 13 | Parent 1: Conrad  Parent 2: Sloan | (Wang et al., 2012A) |
| [Phytoph 15-1](https://www.soybase.org/sbt/search/search_results.php?category=QTLName&search_term=Phytoph+15-1) | 19 | Parent 1: Conrad  Parent 2: Sloan | (Wang et al., 2012B) |
| [Phytoph 15-2](https://www.soybase.org/sbt/search/search_results.php?category=QTLName&search_term=Phytoph+15-2) | 19 | Parent 1: Conrad  Parent 2: Sloan | (Wang et al., 2012B) |
| [Phytoph 15-3](https://www.soybase.org/sbt/search/search_results.php?category=QTLName&search_term=Phytoph+15-3) | 1 | Parent 1: Conrad  Parent 2: Sloan | (Wang et al., 2012B) |
| [Phytoph 15-4](https://www.soybase.org/sbt/search/search_results.php?category=QTLName&search_term=Phytoph+15-4) | 18 | Parent 1: Conrad  Parent 2: Sloan | (Wang et al., 2012B) |
| [Phytoph 15-5](https://www.soybase.org/sbt/search/search_results.php?category=QTLName&search_term=Phytoph+15-5) | 18 | Parent 1: Conrad  Parent 2: Sloan | (Wang et al., 2012B) |

Wang et al. 2012A Comparison of Phenotypic Methods and Yield Contributions of Quantitative Trait Loci for Partial Resistance to *Phyophthora sojae* in Soybean Crop Sci. 2012, 52(2):609-622

Wang et al. 2012B Dissection of two soybean QTL conferring partial resistance to *Phytophthora sojae* through sequence and gene expression analysis BMC Genom. 2012, 13:248
