# Supplementary material for: Genome-Wide Identification of Chalcone Reductase Gene Family in Soybean: Insight into Root-Specific GmCHRs and Phytophthora sojae Resistance
Source: Front Plant Sci. 2017 Dec 7;8:2073. doi: 10.3389/fpls.2017.02073 (PMC5725808; doi:10.3389/fpls.2017.02073)
Supplement: Supplementary file 4 [file Table_3.DOCX]

| QTL Marker  Table S3. List of QTL markers linked to *P sojae* resistance | Chromosome | Location (Glyma 1.0) | Parents | Reference |
| --- | --- | --- | --- | --- |
| Sat_414 | 1 | 51449361 51449420 | Parent 1: Conrad  Parent 2: Sloan | (Wang et al., 2012B) |
| Satt439 | 1 | 48828582 48828608 | Parent 1: OX20-8  Parent 2: PI 398841 | (Lee et al., 2013) |
| Satt266 | 2 | 14090384 14090434 | Parent 1: Conrad  Parent 2: Harosoy | (Burnham et al., 2003) |
| Satt579 | 2 | 19409025 19409087 | Parent 1 : Conrad  Parent 2: Williams | (Burnham et al., 2003) |
| Satt274 | 2 | 48345948 48346001 | Parent 1:Conrad  Parent 2:OX760-6-1 | (Han et al., 2008) |
| Satt373 | 2 | 53.5 cM | Parent 1: OX20-8  Parent 2: PI 398841 | (Lee et al., 2013) |
| Sat_089 | 2 | 37954809 37954854 | Parent 1: Conrad  Parent 2: Hefeng 25 | (Li et al., 2010) |
| Satt600 | 2 | 32526165 32526263 | Parent 1: Conrad  Parent 2: Hefeng 25 | (Li et al., 2010) |
| Satt634 | 2 | 11441849 11441887 | Multiple accession were used | (Sun et al., 2014) |
| Satt542 | 2 | 12956554 12956610 | Multiple accession were used | (Sun et al., 2014) |
| Satt266 | 2 | 14090384 14090434 | Multiple accession were used | (Sun et al., 2014) |
| Sat_423 | 2 | 23541269 23541286 | Multiple accession were used | (Sun et al., 2014) |
| Satt009 | 3 | 3931955 3932116 | Parent 1: OX20-8  Parent 2: PI 398841 | (Lee et al., 2013) |
| ss715585712 | 3 | 3852888 | Multiple accession were used | (Schneider et al., 2016)* |
| ss715585728 | 3 | 3865730 | Multiple accession were used | (Schneider et al., 2016)* |
| ss715586320 | 3 | 4276534 | Multiple accession were used | (Schneider et al., 2016)* |
| ss715586321 | 3 | 4277380 | Multiple accession were used | (Schneider et al., 2016)* |
| ss715586376 | 3 | 4315512 | Multiple accession were used | (Schneider et al., 2016)* |
| Q-03-0266907 | 3 | 36634361 | Multiple accession were used | (Huang et al., 2016)* |
| Q-03-0059953 | 3 | 5147782 | Multiple accession were used | (Huang et al., 2016)* |
| A078_1 | 4 | 985000 4728000 | Parent 1: OX20-8  Parent 2: PI 398841 | (Lee et al., 2013) |
| Satt578 | 4 | 7819442 7819474 | Parent 1: OX20-8  Parent 2: PI 398841 | (Lee et al., 2013) |
| Map-0715 | 4 | 46749591 | Multiple accession were used | (Huang et al., 2016)* |
| Satt100 | 6 | 30668416 30668454 | Parent 1: Conrad  Parent 2: Hefeng 25 | (Li et al., 2010) |
| Satt307 | 6 | 46286881 46286919 | Parent 1: Conrad  Parent 2: Hefeng 25 | (Li et al., 2010) |
| Satt365 | 6 | 111.68 cM | Parent 1: Conrad  Parent 2: Hefeng 25 | (Li et al., 2010) |
| Satt520 | 6 | 7023397 7023432 | Parent 1:Su88-M21(S)  Parent 2: Xinyixiaoheidou (X) | (Wu et al., 2011) |
| AW734043 | 6 | 525,954 526,050 (2) | Parent 1 : Su88-M21  Parent 2 : Xinyixiaoheidou | (Wu et al., 2011) |
| Satt322 | 6 | 12310027 12310059 | Parent 1 : Su88-M21  Parent 2 : Xinyixiaoheidou | (Wu et al., 2011) |
| Satt520 | 6 | 7023397 7023432 | Parent 1 : Su88-M21  Parent 2 : Xinyixiaoheidou | (Wu et al., 2011) |
| Satt557 | 6 | 20018845 20018907 | Parent 1 : Su88-M21  Parent 2 : Xinyixiaoheidou | (Wu et al., 2011) |
| Satt079 | 6 | 43950980 43951015 | Parent 1 : Su88-M21  Parent 2 : Xinyixiaoheidou | (Wu et al., 2011) |
| Satt307 | 6 | 46286881 46286919 | Parent 1 : Su88-M21  Parent 2 : Xinyixiaoheidou | (Wu et al., 2011) |
| Satt316 | 6 | 47485158 47485193 | Parent 1 : Su88-M21  Parent 2 : Xinyixiaoheidou | (Wu et al., 2011) |
| Satt376 | 6 | 15482894 15482935 | Parent 1 : Su88-M21  Parent 2 : Xinyixiaoheidou | (Wu et al., 2011) |
| Staga001 | 6 | 45995029 | Parent 1 : Su88-M21  Parent 2 : Xinyixiaoheidou | (Wu et al., 2011) |
| Sat_251 | 6 | 39010070 39010131 | Parent 1 : Su88-M21  Parent 2 : Xinyixiaoheidou | (Wu et al., 2011) |
| Sat_246 | 6 | 14444925 14444988 | Parent 1 : Su88-M21  Parent 2 : Xinyixiaoheidou | (Wu et al., 2011) |
| BARC-014527-01571 | 6 | 644565 | Multiple accession were used | (Huang et al., 2016)* |
| Satt463 | 7 | 8243902 8244048 | Parent 1: OX20-8  Parent 2: PI 398841 | (Lee et al., 2013) |
| Satt437 | 8 | 18925608 18925978 | Parent 1: Conrad  Parent 2: Hefeng 25 | (Li et al., 2010) |
| GMA2_OSU19 | 8 | 5526515 | Parent 1: Conrad  Parent 2: Sloan | (Wang et al., 2012A) |
| Satt632 | 8 | 8219301 8219351 | Parent 1: Conrad  Parent 2: Sloan | (Wang et al., 2012A) |
| Map-1630 | 9 | 3157784 | Multiple accession were used | (Huang et al., 2016)* |
| Sat_274 | 10 | 43209696 43209751 | Parent 1:Su88-M21(S)  Parent 2: Xinyixiaoheidou (X) | (Wu et al., 2011) |
| Satt358 | 10 | 1022833 1022889 | Parent 1 : Su88-M21  Parent 2 : Xinyixiaoheidou | (Wu et al., 2011) |
| Sat_321 | 10 | 2471412 2471651 | Parent 1 : Su88-M21  Parent 2 : Xinyixiaoheidou | (Wu et al., 2011) |
| Satt445 | 10 | 2654084 2654152 | Parent 1 : Su88-M21  Parent 2 : Xinyixiaoheidou | (Wu et al., 2011) |
| Satt241 | 10 | 32898701 32898763 | Parent 1 : Su88-M21  Parent 2 : Xinyixiaoheidou | (Wu et al., 2011) |
| Satt345 | 10 | 12172070 12172150 | Parent 1 : Su88-M21  Parent 2 : Xinyixiaoheidou | (Wu et al., 2011) |
| Satt094 | 10 | 12407171 12407229 | Parent 1 : Su88-M21  Parent 2 : Xinyixiaoheidou | (Wu et al., 2011) |
| Satt550 | 10 | 17887640 17887687 | Parent 1 : Su88-M21  Parent 2 : Xinyixiaoheidou | (Wu et al., 2011) |
| Satt576 | 10 | 12566766 12567052 | Parent 1 : Su88-M21  Parent 2 : Xinyixiaoheidou | (Wu et al., 2011) |
| Satt479 | 10 | 28671499 28671546 | Parent 1 : Su88-M21  Parent 2 : Xinyixiaoheidou | (Wu et al., 2011) |
| Satt188 | 10 | 29656018 29656110 | Parent 1 : Su88-M21  Parent 2 : Xinyixiaoheidou | (Wu et al., 2011) |
| Satt420 | 10 | 9907940 9907987 | Parent 1 : Su88-M21  Parent 2 : Xinyixiaoheidou | (Wu et al., 2011) |
| Sat_242 | 10 | 38844768 38844803 | Parent 1 : Su88-M21  Parent 2 : Xinyixiaoheidou | (Wu et al., 2011) |
| Satt592 | 10 | 42983859 4298389 | Parent 1 : Su88-M21  Parent 2 : Xinyixiaoheidou | (Wu et al., 2011) |
| Sat_196 | 10 | 179136 179191 | Parent 1 : Su88-M21  Parent 2 : Xinyixiaoheidou | (Wu et al., 2011) |
| Sat_273 | 10 | 10403235 10403288 | Parent 1 : Su88-M21  Parent 2 : Xinyixiaoheidou | (Wu et al., 2011) |
| Satt453 | 11 | 38360612 38360653 | Parent 1: Conrad  Parent 2: Hefeng 25 | (Li et al., 2010) |
| Map-1995 | 11 | 7904934 | Multiple accession were used | (Huang et al., 2016)* |
| A036_1 | 12 | 34.29 cM | Parent 1: Conrad  Parent 2: Sloan | Wang et al. 2010 |
| GMH_OSU31 | 12 | 5526515 | Parent 1: Conrad  Parent 2: Sloan | (Wang et al., 2012A) |
| Satt252 | 13 | 5376564 5376632 | Parent 1: Conrad  Parent 2: Harosoy | (Burnham et al., 2003) |
| Satt030 | 13 | 8722687 8722749 | Parent 1:Conrad  Parent 2:OX760-6-1 | (Han et al., 2008) |
| Satt343 | 13 | 11494473 11494511 | Parent 1:Conrad  Parent 2:OX760-6-1 | (Han et al., 2008) |
| Sat_133 | 13 | 23462623 23462676 | Parent 1: OX20-8  Parent 2: PI 398841 | (Lee et al., 2013) |
| Satt343 | 13 | 11494473 11494511 | Parent 1: Conrad  Parent 2: Hefeng 25 | (Li et al., 2010) |
| Sct_033 | 13 | 30739608 30739666 | Parent 1: S99-2281  Parent 2: PI 408105A | (Nguyen et al., 2012) |
| K644_1 | 13 | 67.18 cM | Parent 1: V71-370  Parent 2: PI407162 | (Tucker et al., 2010) |
| Satt160 | 13 | 3993326 3993415 | Parent 1: Conrad  Parent 2: Sloan | Wang et al. 2010 |
| F424_294 | 13 | 33.2 cM | Parent 1: Conrad  Parent 2: Sloan | (Wang et al., 2012A) |
| ss715615031 | 13 | 30766058 | Multiple accession were used | (Schneider et al., 2016)* |
| Satt304 | 14 | 13284499 13284588 | Parent 1: Conrad  Parent 2: Sloan | Wang et al. 2010 |
| Satt384 | 15 | 4036564 4036611 | Parent 1: OX20-8  Parent 2: PI 398841 | (Lee et al., 2013) |
| Satt651 | 15 | 6805203 6805232 | Parent 1:Su88-M21(S)  Parent 2: Xinyixiaoheidou (X) | (Wu et al., 2011) |
| Satt369 | 15 | 48217766 48217816 | Parent 1 : Su88-M21  Parent 2 : Xinyixiaoheidou | (Wu et al., 2011) |
| Satt204 | 15 | 17735000 17735057 | Parent 1 : Su88-M21  Parent 2 : Xinyixiaoheidou | (Wu et al., 2011) |
| Satt491 | 15 | 43.64 cM | Parent 1 : Su88-M21  Parent 2 : Xinyixiaoheidou | (Wu et al., 2011) |
| Satt268 | 15 | 22885468 22885518 | Parent 1 : Su88-M21  Parent 2 : Xinyixiaoheidou | (Wu et al., 2011) |
| Sat_380 | 15 | 17025462 17025485 | Parent 1 : Su88-M21  Parent 2 : Xinyixiaoheidou | (Wu et al., 2011) |
| Satt598 | 15 | 13638366 13638395 | Parent 1 : Su88-M21  Parent 2 : Xinyixiaoheidou | (Wu et al., 2011) |
| Satt384 | 15 | 4036564 4036611 | Parent 1 : Su88-M21  Parent 2 : Xinyixiaoheidou | (Wu et al., 2011) |
| Satt720 | 15 | 12310027 12310059 | Parent 1 : Su88-M21  Parent 2 : Xinyixiaoheidou | (Wu et al., 2011) |
| Satt403 | 15 | 44.93 cM | Parent 1 : Su88-M21  Parent 2 : Xinyixiaoheidou | (Wu et al., 2011) |
| Q-15-0369188 | 15 | 48863575 | Multiple accession were used | (Huang et al., 2016)* |
| Q-15-0128012 | 15 | 15532390 | Multiple accession were used | (Huang et al., 2016)* |
| BARC-039153-07459 | 16 | 3962328 | Multiple accession were used | (Huang et al., 2016)* |
| Satt414 | 16 | 37.04 cM | Parent 1: V71-370  Parent 2: PI407162 | (Tucker et al., 2010) |
| Satt596 | 16 | 39. 63 cM | Parent 1: Conrad  Parent 2: OX760-6-1 | (Weng et al., 2007) |
| Q-16-0268535 | 16 | 33793393 | Multiple accession were used | (Huang et al., 2016)* |
| BARC-014467-01559 | 16 | 3962328 | Multiple accession were used | (Huang et al., 2016)* |
| Map-3031 | 16 | 15093996 | Multiple accession were used | (Huang et al., 2016)* |
| BARC-042413-08254 | 16 | 35175092 | Multiple accession were used | (Huang et al., 2016)* |
| Satt543 | 17 | 30637442 30637498 | Parent 1: S99-2281  Parent 2: PI 408105A | (Nguyen et al., 2012) |
| Satt_222 | 17 | 68.08 cM | Multiple accession were used | (Sun et al., 2014) |
| Satt226 | 17 | 26354609 26354928 | Multiple accession were used | (Sun et al., 2014) |
| Satt300 | 17 | 30.93 cM | Multiple accession were used | (Sun et al., 2014) |
| Satt574 | 17 | 31915278 31915313 | Multiple accession were used | (Sun et al., 2014) |
| Satt543 | 17 | 30637442 30637498 | Multiple accession were used | (Sun et al., 2014) |
| Satt615 | 17 | 91.2 cM | Multiple accession were used | (Sun et al., 2014) |
| Satt301 | 17 | 37009236 37009310 | Multiple accession were used | (Sun et al., 2014) |
| Satt574 | 17 | 31915278 31915313 | Parent 1: Conrad  Parent 2: Sloan | (Wang et al. 2010) |
| OPAD08 | 18 | 93cM | Parent 1: OX20-8  Parent 2: PI 398841 | (Lee et al., 2013) |
| Satt688 | 18 | 3264343 3264375 | Parent 1: V71-370  Parent 2: PI407162 | (Tucker et al., 2010) |
| A681_1 | 18 | 116.76 cM | Parent 1: Conrad  Parent 2: Sloan | (Wang et al., 2012B) |
| Satt472 | 18 | 53866536 53866808 | Parent 1: Conrad  Parent 2: Sloan | (Wang et al., 2012B) |
| BARC-039397-07314 | 18 | 56889971 56889971 | Parent 1: Conrad  Parent 2: Sloan | (Wang et al., 2012B) |
| BARCSOYSSR_18_1707 | 18 | 58122596 58122639  53852971 53853014 (2) | Parent 1: Conrad  Parent 2: Sloan | (Wang et al., 2012B) |
| BARCSOYSSR_18_1777 | 18 | 59016077 59016134  54744147 54744204 (2) | Parent 1: Conrad  Parent 2: Sloan | (Wang et al., 2012B) |
| BARCSOYSSR_19_1393 | 19 | 46388480 4,388539 | Parent 1: Conrad  Parent 2: Sloan | (Wang et al., 2012A) |
| GM19_OSU10 | 18 | 28.8 cM | Parent 1: Conrad  Parent 2: Sloan | (Wang et al., 2012A) |
| Satt527 | 19 | 42835228 42835278 | Parent 1: Conrad  Parent 2: Sloan | Wang et al. 2010  Geno. 2010, 3(1):23-40 |
| BARC-021321-04035 | 19 | 4737800 147378001 | Parent 1: Conrad  Parent 2: Sloan | (Wang et al., 2012A) |
| Satt527 | 19 | 42835228 42835278 | Parent 1: Conrad  Parent 2: Sloan | (Wang et al., 2012B) |
| BARC-03997-07624 | 19 | 74.1 cM | Parent 1: Conrad  Parent 2: Sloan | (Wang et al., 2012A) |
| BARC-064609-18739 | 19 | 47232960 47232960 | Parent 1: Conrad  Parent 2: Sloan | (Wang et al., 2012A) |
| BARCSOYSSR_18_1793 | 19 | 55001577 55001686 | Parent 1: Conrad  Parent 2: Sloan | (Wang et al., 2012A) |
| BARCSOYSSR_19_1243 | 19 | 43330107 43330174  43533689 43533756 (2) | Parent 1: Conrad  Parent 2: Sloan | (Wang et al., 2012B) |
| BARCSOYSSR_19_1473 | 19 | 47801595 47801650  47923547 47923602 (2) | Parent 1: Conrad  Parent 2: Sloan | (Wang et al., 2012B) |
| BARCSOYSSR_19_1494 | 19 | 48338071 48338124 | Parent 1: Conrad  Parent 2: Sloan | (Wang et al., 2012A) |
| BARCSOYSSR_19_1532 | 19 | 49060024 49060065 | Parent 1: Conrad  Parent 2: Sloan | (Wang et al., 2012A) |
| GML_OSU42 | 19 | 44019432 | Parent 1: Conrad  Parent 2: Sloan | (Wang et al., 2012A) |
| ss715635897 | 19 | 49121258 | Multiple accession were used | (Schneider et al., 2016)* |
| ss715635934 | 19 | 49461582 | Multiple accession were used | (Schneider et al., 2016)* |
| ss715636056 | 19 | 50544363 | Multiple accession were used | (Schneider et al., 2016)* |
| ss715636059 | 19 | 50555433 | Multiple accession were used | (Schneider et al., 2016)* |
| ss715636064 | 19 | 50604933 | Multiple accession were used | (Schneider et al., 2016)* |
| ss715636073 | 19 | 50663466 | Multiple accession were used | (Schneider et al., 2016)* |
| ss715636076 | 19 | 50666563 | Multiple accession were used | (Schneider et al., 2016)* |
| ss715636077 | 19 | 50668662 | Multiple accession were used | (Schneider et al., 2016)* |
| ss715636083 | 19 | 50679714 | Multiple accession were used | (Schneider et al., 2016)* |
| ss715636084 | 19 | 50681263 | Multiple accession were used | (Schneider et al., 2016)* |
| Sat_268 | 20 | 35176184 3517624 | Parent 1: OX20-8  Parent 2: PI 398841 | (Lee et al., 2013) |
| BARC-013645-01207 | 20 | 46624541 | Multiple accession were used | (Huang et al., 2016)* |
| Satt614 | 20 | 3915962 3916075 | V71-370  PI407162 | (Tucker et al., 2010) |

References

**Burnham K, Dorrance A, VanToai T, St Martin S** (2003) Quantitative Trait Loci for Partial Resistance to in Soybean. Crop science **43:** 1610-1617

**Han Y, Teng W, Yu K, Poysa V, Anderson T, Qiu L, Lightfoot D, Li W** (2008) Mapping QTL tolerance to Phytophthora root rot in soybean using microsatellite and RAPD/SCAR derived markers. Euphytica **162:** 231-239

**Lee S, Mian MAR, McHale LK, Wang H, Wijeratne AJ, Sneller CH, Dorrance AE** (2013) Novel quantitative trait loci for partial resistance to Phytophthora sojae in soybean PI 398841. TAG. Theoretical and Applied Genetics. Theoretische Und Angewandte Genetik **126:** 1121-1132

**Li XP, Han YP, Teng WL, Zhang SZ, Yu KF, Poysa V, Anderson T, Ding JJ, Li WB** (2010) Pyramided QTL underlying tolerance to Phytophthora root rot in mega-environments from soybean cultivars 'Conrad' and 'Hefeng 25'. Theoretical and Applied Genetics **121:** 651-658

**Nguyen V, Vuong T, VanToai T, Lee J, Wu X, Mian M, Dorrance A, Shannon J, Nguyen H** (2012) Mapping of quantitative trait loci associated with resistance to and flooding tolerance in soybean. Crop Science **52:** 2481-2493

**Sun J, Guo N, Lei J, Li L, Hu G, Xing H** (2014) Association mapping for partial resistance to Phytophthora sojae in soybean (Glycine max (L.) Merr.). Journal of genetics **93:** 355-363

**Tucker DM, Saghai Maroof MA, Mideros S, Skoneczka JA, Nabati DA, Buss GR, Hoeschele I, Tyler BM, St. Martin SK, Dorrance AE** (2010) Mapping Quantitative Trait Loci for Partial Resistance to in a Soybean Interspecific Cross. Crop Science **50:** 628-635

**Wang H, St Martin SK, Dorrance AE** (2012A) Comparison of Phenotypic Methods and Yield Contributions of Quantitative Trait Loci for Partial Resistance to in Soybean. Crop Science **52:** 609-622

**Wang H, Wijeratne A, Wijeratne S, Lee S, Taylor CG, St Martin SK, McHale L, Dorrance AE** (2012B) Dissection of two soybean QTL conferring partial resistance to Phytophthora sojae through sequence and gene expression analysis. BMC Genomics **13:** 428

**Weng C, Yu K, Anderson T, Poysa V** (2007) A quantitative trait locus influencing tolerance to Phytophthora root rot in the soybean cultivar ‘Conrad’. Euphytica **158:** 81-86

**Wu X, Zhou B, Zhao J, Guo N, Zhang B, Yang F, Chen S, Gai J, Xing H** (2011) Identification of quantitative trait loci for partial resistance to Phytophthora sojae in soybean. Plant Breeding **130:** 144-149
